# The Fgf8 subfamily (Fgf8, Fgf17 and Fgf18) is required for closure of the embryonic ventral body wall

**DOI:** 10.1242/dev.189506

**Published:** 2020-10-19

**Authors:** Michael Boylan, Matthew J. Anderson, David M. Ornitz, Mark Lewandoski

**Affiliations:** 1 Cancer and Developmental Biology Lab, National Cancer Institute, National Institutes of Health, Frederick, MD 21702, USA; 2 Department of Developmental Biology, Washington University School of Medicine, Saint Louis, MO 63110, USA

**Keywords:** Omphalocele, Fibroblast growth factor, Ventral body wall

## Abstract

The closure of the embryonic ventral body wall in amniotes is an important morphogenetic event and is essential for life. Defects in human ventral wall closure are a major class of birth defect and a significant health burden. Despite this, very little is understood about how the ventral body wall is formed. Here, we show that fibroblast growth factor (FGF) ligands FGF8, FGF17 and FGF18 are essential for this process. Conditional mouse mutants for these genes display subtle migratory defects in the abdominal muscles of the ventral body wall and an enlarged umbilical ring, through which the internal organs are extruded. By refining where and when these genes are required using different Cre lines, we show that *Fgf8* and *Fgf17* are required in the presomitic mesoderm, whereas *Fgf18* is required in the somites. This study identifies complex and multifactorial origins of ventral wall defects and has important implications for understanding their origins during embryonic development.

## Abstract (precis)

**Summary:** This study demonstrates that three related FGFs (FGF8, FGF17 and FGF18) cooperate in the essential, but understudied, process of embryonic ventral body wall closure in amniotes.

## INTRODUCTION

Embryonic ventral wall (VW) defects are a class of congenital abnormality and are relatively frequently encountered in the clinic. Omphalocele is a VW defect where the viscera are herniated through an enlarged umbilical ring. Usually, the organs remain covered by the amnion (Williams, 2008). Severity can range from only a portion of liver being herniated to the extrusion of multiple organs. Omphalocele is frequently co-morbid with other defects, especially cardiac defects, pulmonary hypertension and chromosomal abnormalities (Corey et al., 2014; Marshall et al., 2015), and these contribute to the high perinatal mortality rate. Omphalocele is sometimes confused with another VW defect, gastroschisis, and this confusion has been noted in the literature (Carnaghan et al., 2013; Williams, 2008). In omphalocele, the defect is centered on the umbilical ring and the viscera are contained within the amniotic membrane unless the membrane has ruptured (Brewer and Williams, 2004a). In gastroschisis the defect is dextral to the umbilical ring, usually only loops of midgut are herniated, and the defect is never covered by a membrane (Brewer and Williams, 2004a).

Despite its medical relevance, VW closure is poorly understood. The VW has two components, the primary and secondary VW, both derived from the embryonic mesoderm emerging from the primitive streak. The primary VW is the initial covering for the ventral surface, and, in the mouse, is formed at embryonic day (E) 9.5 by the midline fusion of the left and right halves of the lateral plate mesoderm (LPM). At around E11.0, muscle, tendon and cartilage progenitors from the somites migrate ventrolaterally (Nichol et al., 2012); these will form the abdominal muscles, connective tissues and ribs, respectively, that comprise the secondary VW. Migration is complete by E14.5, but the midgut, within the physiological hernia, still protrudes from the embryo through the umbilical ring, where the umbilical vessels connect into the embryo. The midgut returns to the abdomen by E16.5. In human omphalocele, migration of abdominal wall muscles appears immature and disorganized (Nichol et al., 2012), leading to the hypothesis that defects in muscle migration causes omphalocele in both mice and humans. However, many mouse models of muscle defects have been described that do not report VW defects (Grifone et al., 2005; Rudnicki et al., 1993; Tremblay et al., 1998), making the causative relationship between secondary VW migration and omphalocele unclear.

The fibroblast growth factor (FGF) pathway is one of the cardinal cell signaling pathways in embryology, with 18 secreted signaling ligands grouped into seven subfamilies based on their sequence homology (Ornitz and Itoh, 2015). *Fgf8* is expressed early in embryogenesis (Crossley and Martin, 1995) and is essential for the morphogenesis of many tissues including the kidneys (Perantoni et al., 2005), limbs (Crossley et al., 1996; Lewandoski et al., 2000) and others, and is also essential for gastrulation (Sun et al., 1999). Based on amino acid sequence similarity, there are two other members of the *Fgf8* subfamily, *Fgf17* and *Fgf18*, with unique expression patterns during embryonic development (Maruoka et al., 1998; Xu et al., 1999). *Fgf17* plays a role in brain development (Cholfin and Rubenstein, 2007; Xu et al., 1999), and although *Fgf17* null mice are viable, they exhibit subtle behavioral abnormalities (Scearce-Levie et al., 2008). *Fgf18* is important in regulating chondrogenesis and osteogenesis (Hung et al., 2016; Liu et al., 2007, 2002; Ohbayashi et al., 2002)*. Fgf8* expression in the presomitic mesoderm (PSM) plays a role in the wavefront of somitogenesis, an activity that prevents differentiation of this tissue (Dubrulle and Pourquié, 2004; Naiche et al., 2011). *Fgf17* and *Fgf18* are also expressed in the PSM (Maruoka et al., 1998), but a functional PSM role is unknown for these two genes. Owing to their ability to genetically compensate for one another, FGF ligand requirements in any morphogenetic processes can be obscured until multiple ligands are inactivated (Naiche et al., 2011). We decided to search for genetic interactions between the members of the *Fgf8* subfamily, *Fgf8*, *Fgf17* and *Fgf18*, within the PSM. We found that when all three genes are conditionally inactivated, we recovered embryos with omphalocele.

Other signaling pathways have been implicated in controlling VW morphogenesis including the Hedgehog (Matsumaru et al., 2014), TGFβ (Aldeiri et al., 2017), PCP (Murdoch et al., 2014) and canonical Wnt (Zhang et al., 2014) pathways. Inactivating some downstream components of the FGF pathway has been shown to result in omphalocele. Inactivating of one copy of *Fgfr1* and both copies of *Fgfr2* using a global inducible Cre causes omphalocele (Nichol et al., 2011). Similarly, inactivating *Mek1* (also known as *Map2k1*) in a *Mek2* (*Map2k2*) null background with a mesenchyme-specific Cre resulted in omphalocele (Boucherat et al., 2014). Here, we investigate the genetic interaction between different members of the *Fgf8* subfamily and examine their roles in VW morphogenesis. By using different tissue-specific Cre recombinase mouse lines, we demonstrate that the requirement for these genes in VW morphogenesis is in the PSM and somites. This is the first evidence connecting a loss of FGF ligand function to VW defects.

## RESULTS

### The Fgf8 subfamily is expressed in progenitors of the primary and secondary body wall

**Fig. 1.**
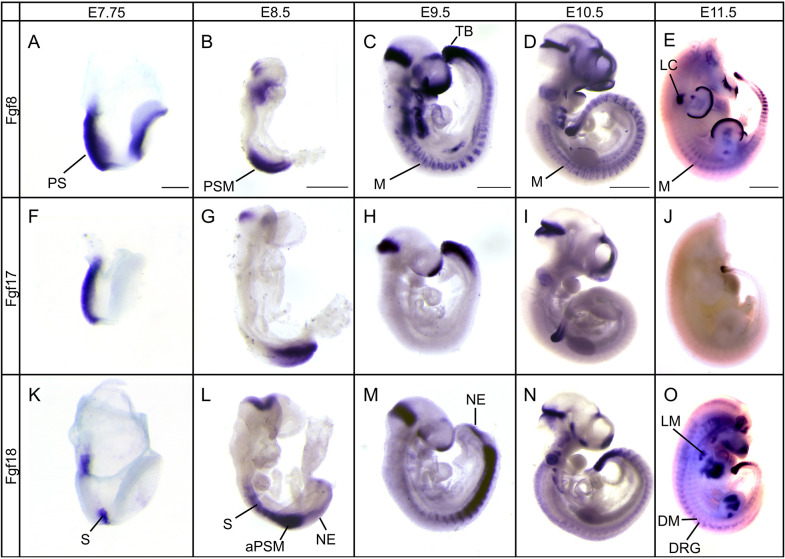
**Members of the *Fgf8* subfamily are expressed in multiple overlapping domains.** (A-E) Expression pattern of *Fgf8* from E7.75 to E11.5. (F-J) Expression pattern of *Fgf17* from E7.75 to E11.5. (K-O) Expression pattern of *Fgf18* from E7.75 to E11.5. E11.5 embryos were decapitated before WISH to reduce probe trapping. All three genes are expressed in and around the PSM, and *Fgf8* and *Fgf18* are expressed in the somites and the dermomyotome and myotome. aPSM, anterior presomitic mesoderm; DM, dermomyotome; DRG, dorsal root ganglion; LC, limb bud condensation; LM, limb muscle; M, myotome; NE, neuroepithelium; PS, primitive streak; PSM, presomitic mesoderm; S, somite; TB, tail bud. Scale bars: 250 μm (A); 500 μm (B,C); 1 mm (D,E).

We first examined the expression patterns of *Fgf8* (Fig. 1A-E), *Fgf17* (Fig. 1F-J) and *Fgf18* (Fig. 1K-O) from E7.75 through to E11.5 using RNA whole-mount *in situ* hybridization reaction (WISH). We developed the chromogenic stain for an extended period of time to reveal domains of low gene expression. Both *Fgf8* and *Fgf17* were expressed in the primitive streak, tailbud and the PSM (Fig. 1A-J). *Fgf8* (Fig. 1C-E) was expressed in the myotome of the somites, whereas *Fgf18* (Fig. 1K-O) was expressed more broadly in somites. In addition to these observations, which replicate previously published expression patterns (Crossley and Martin, 1995; Hagan et al., 2019; Maruoka et al., 1998; Stolte et al., 2002), we report previously undescribed expression patterns for *Fgf8* and *Fgf18*. At E11.5, *Fgf8* was weakly expressed in the myotome in the interlimb region, as well as in the condensing portions of the limb (Fig. 1E). *Fgf18* was expressed in the neuroepithelium of the tailbud from E8.5 through E10.5 (Fig. 1L-O) and in the dermomyotome at E11.5 (Fig. 1O).

*Fgf8* and *Fgf17* are expressed in the primitive streak and PSM, which gives rise to the LPM and ultimately to the primary VW; the PSM also forms the somites, which generate the secondary VW. *Fgf8* and *Fgf18* are also expressed in the somites themselves. These experiments demonstrate that these Fgfs are expressed in the progenitors of the embryonic structures that will close the VW. The data in Fig. 1 indicate that there is either no or very low expression of these ligand genes in the primary VW or secondary VW layers themselves. To address this directly, we generated mRNA E11.5 *in situ* hybridization chain reaction (HCR) data for each of the three FGF ligand genes (Fig. S1). These analyses confirmed our observation that there is little to no detectable expression within the VW itself.

### The Fgf8 subfamily is required to close the ventral body wall

Inactivation of *Fgf8* in the nascent mesoderm, emerging from the primitive streak via Tg(T-cre)1Lwd (hereafter ‘TCre’) transgenic activity, demonstrated its role in nephrogenesis (Perantoni et al., 2005) and the male urogenital tract (Kitagaki et al., 2011). Inactivation of *Fgf8* simultaneously with *Fgf4* using TCre showed that these FGFs redundantly maintain the PSM in an undifferentiated state (Naiche et al., 2011). Therefore, we asked whether there were redundant roles for the *Fgf8* subfamily in the nascent mesoderm and its derivatives. To address this, we conditionally inactivated *Fgf8* and *Fgf18* in the primitive steak using TCre on an *Fgf17* null background (Table S1). We confirmed that TCre recombines in the primitive streak and thus throughout most of the mesoderm by breeding TCre males to *Gt(ROSA)26Sor^tm4(ACTB-tdTomato,-EGFP)Luo^*/J (hereafter ‘*mTmG*’) reporter females (Muzumdar et al., 2007) (Fig. S2A). We confirmed that both floxed genes were recombined using WISH analysis with riboprobes directed against the deleted region of each gene and observed that expression of *Fgf8* and *Fgf18* was abolished in tissues recombined by Cre recombinase (Fig. S2B-E).

**Fig. 2.**
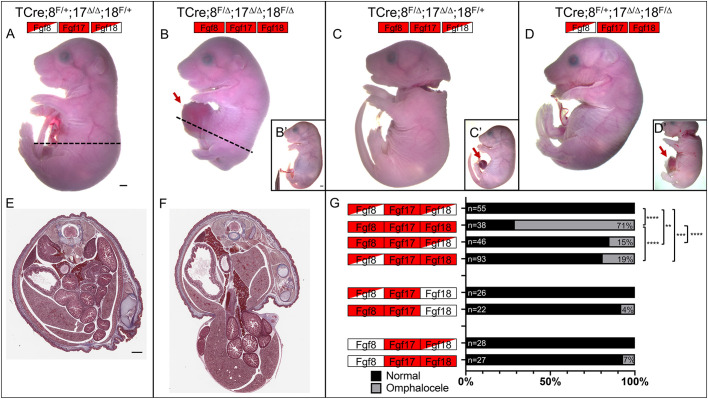
**E18.5 *Fgf8* subfamily triple mutants have omphalocele.** (A) Control embryos appear phenotypically normal. (B) Most triple mutants have omphalocele (arrow), though a subset do not (B′). (C) TCre*;Fgf8^f/Δ^,17^Δ/Δ^,18^f/+^* embryos usually appear phenotypically normal, but a subset have omphalocele (C′). (D) TCre*;Fgf8^f/+^,17^Δ/Δ^,18^f/Δ^* offspring have kyphosis and bowed limbs; a minority of embryos also have omphalocele (D′). (E,F) transverse sections of control (E) and triple mutant (F) embryos stained with Masson's trichrome. The axial level of the section is indicated by the dashed line in A and B. The omphalocele is within an amniotic sac, and all five layers of muscle are present, albeit thinner (*n*=4). (G) Graphical representation of the incidence of omphalocele showing the percentage of embryos with and without omphalocele. The total number of embryos is to the left of the bars and the percentage of embryos with omphalocele is on the right. Significance was determined using a two-tailed Fisher's exact test. **P<0.01, ***P<0.001, ****P<0.0001. Boxes indicate genotype: an empty box indicates two wild-type alleles; a half red, half empty box indicates one mutant and one wild-type allele; a fully red box indicates two mutant alleles. Scale bars: 1 mm.

At E18.5 TCre*;Fgf8^f/+^;Fgf17^Δ/Δ^;Fgf18^f/+^* offspring (hereafter ‘controls’) were healthy and viable with no obvious abnormal phenotype (Fig. 2A). However, when we generated TCre*;Fgf8^f/Δ^;Fgf17^Δ/Δ^;Fgf18^f/Δ^* littermates (hereafter ‘triple mutants’) we found that these embryos frequently had omphalocele (Fig. 2B), with 71% penetrance (Fig. 2G). This failure to close the ventral body was always abdominal and never affected the thoracic body wall. TCre*;Fgf8^f/Δ^;Fgf17^Δ/Δ^;Fgf18^f/+^* offspring (Fig. 2C) occasionally had omphalocele (Fig. 2C′,G) but otherwise resembled TCre*;Fgf8^f/Δ^* animals (Kitagaki et al., 2011; Perantoni et al., 2005). TCre*;Fgf8^f/+^;Fgf17^Δ/Δ^;Fgf18^f/Δ^* embryos (Fig. 2D) also occasionally presented with omphalocele (Fig. 2D′,G), but otherwise displayed no additional phenotype beyond the kyphosis and other skeletal defects already reported for TCre*;Fgf18^f/Δ^* animals (Hagan et al., 2019) (Fig. S3C,G). We observed that embryos were present in non-Mendelian ratios, with triple mutants appearing underrepresented (*P*<0.02, two-tailed Chi^2^ test) compared with controls. As described below, this is likely because a subset of these mutants failed to undergo proper yolk sac development.

In triple mutant embryos the omphalocele ranged in severity, from only a portion of the liver being herniated, to most of the liver as well as the large and small intestine and the stomach being herniated. The amnion around the hernia indicated that the defect was omphalocele, not gastroschisis. Sections revealed that there were no gross morphological defects in the abdominal muscles at E18.5 in triple mutants (*n*=4), with all the muscle layers present and morphologically normal (Fig. 2E,F). The skeletal defects attributable to a loss of *Fgf18* (Hagan et al., 2019; Hung et al., 2016; Liu et al., 2007, 2002; Ohbayashi et al., 2002) in the triple mutants were more severe than in TCre*;Fgf8^f/+^;Fgf17^Δ/Δ^;Fgf18^f/Δ^* embryos (Fig. S3D,H). In triple mutants the kyphosis was more pronounced and the ribs more bowed than in TCre*;Fgf8^f/+^;Fgf17^Δ/Δ^;Fgf18^f/Δ^* embryos (compare Fig. S3C,G with D,H). Although the sternum appeared kinked because of the rib abnormalities, it had fused correctly (Fig. S3D′). No other abnormalities that are often associated with VW defects, such as genitourinary deformities (Matsumaru et al., 2014) or diaphragmatic hernia (Stoll et al., 2008), were observed.

We then proceeded to test the dosage requirements for each gene in recovering omphalocele by performing a genetic series in which different members of the *Fgf8* subfamily are inactivated in different combinations. We observed that in the triple mutants, the recovery rate of omphalocele was 71% (Fig. 2G); when both copies of *Fgf17* are intact in a TCre*;Fgf8^f/Δ-^;Fgf18^f/Δ^* background this rate was reduced to 52% (Fig. S4), suggesting a role for *Fgf17*, although this reduction was not significant (*P*=0.1733, two-tailed Fisher's exact test). To analyze the role of *Fgf17* further we considered double mutants in which *Fgf17* was inactivated along with either *Fgf8* or *Fgf18*. In such mutants (TCre*;Fgf8^f/Δ^;Fgf17^Δ/Δ^;Fgf18^+/+^* and TCre*;Fgf8^+/+^;Fgf17^Δ/Δ^;Fgf18^f/Δ^*) the incidence of omphalocele was 4% and 7%, respectively (Fig. 2G). When only *Fgf8* (*n*=18) or *Fgf18* (*n*=30) were inactivated via TCre-mediated recombination, omphalocele never occurred, demonstrating a small but real effect from the loss of *Fgf17*. Having established that all three genes play a role, we continued the analysis in an *Fgf17* null background.

### Omphalocele occurs between E12.5 and E13.5 and is associated with defects in the structures of the body wall

**Fig. 3.**
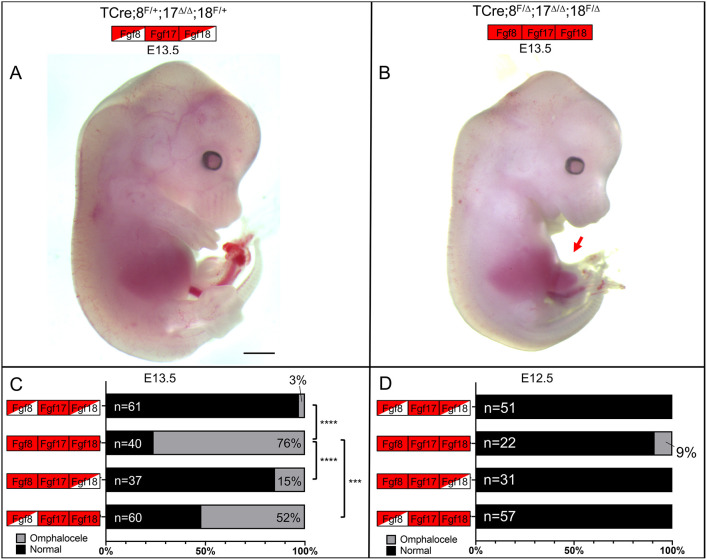
**The omphalocele phenotype in triple mutants is evident at E13.5.** (A) E13.5 control embryo. (B) E13.5 triple mutant embryo with an abnormal inclusion of liver in the physiological hernia (arrow), indicating omphalocele. (C,D) Graphical representation of the incidence of omphalocele at E13.5 (C) and E12.5 (D) showing the percentage of embryos with and without omphalocele. The total number of embryos is to the left of the bars and the percentage of embryos with omphalocele is on the right. Significance was determined using a two-tailed Fisher's exact test. ***P<0.001, ****P<0.0001. Boxes indicate genotype; see Fig. 2 legend for key. Scale bar: 1 mm.

To determine when omphalocele occurs, we performed timed dissections at E12.5 and E13.5. At E13.5, the phenotype was fully evident (Fig. 3A-C), with the rate of occurrence equivalent to E18.5 (76% versus 71%, respectively). At this developmental stage, the only morphological evidence of omphalocele was the aberrant presence of liver in the physiological hernia (Fig. 3B, red arrow). Embryos were recorded as positive for omphalocele if any piece of liver was present. If these small extrusions of liver into the physiological hernia returned to the abdomen, the incidence of omphalocele would be overcounted at E13.5 compared with E18.5, which could account for the apparent increase in omphalocele at E13.5 in both controls and in TCre*;Fgf8^f/+^;Fgf17^Δ/Δ^;Fgf18^f/Δ^* embryos (compare Fig. 2G with Fig. 3C). At E12.5, omphalocele was infrequently observed (9%) in triple mutants, and never observed in any other genotype (Fig. 3D). From this we concluded that omphalocele has not yet occurred at E12.5 in the vast majority of embryos.

We also examined earlier embryonic stages. At E10.5 in TCre*;Fgf8^f/Δ^;Fgf17^Δ/Δ^;Fgf18^f/+^* embryos and in triple mutants, a significant subset had failed to undergo proper yolk sac development (Fig. S5A,B). The incidence of defects at E10.5 trended higher in triple mutant embryos compared with TCre*;Fgf8^f/Δ^;Fgf17^Δ/Δ^;Fgf18^f/+^* embryos (Fig. S5C), suggesting that the loss of *Fgf18* increases the penetrance of the yolk sac defects. This explains why there are fewer embryos of these genotypes at E18.5, as some of these embryos would have died *in utero* (Fig. 2G).

**Fig. 4.**
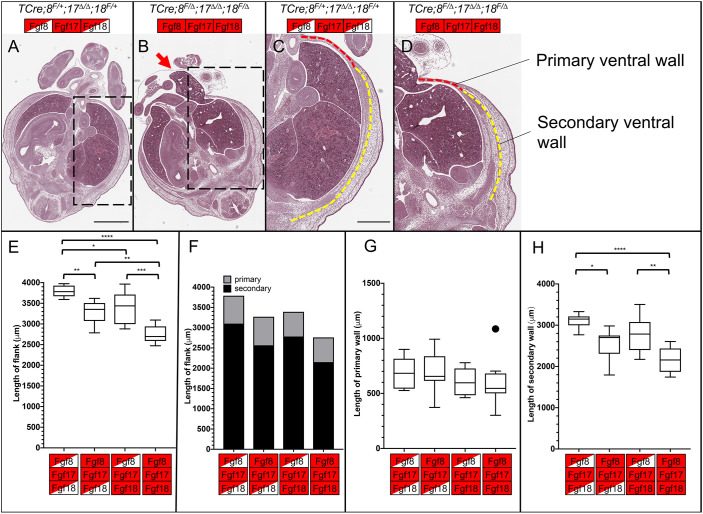
**Morphometric analysis of sections at E13.5 reveals changes in the secondary** **VW** **of *Fgf8* subfamily mutants.** (A,B) Transverse section of E13.5 control (A) and triple mutant (B) at the interlimb region. Note the inclusion of liver in the physiological hernia of the triple mutant (arrow). (C,D) Enlargement of boxed regions of A (C) and B (D) showing the primary (red dashed line) and secondary (yellow dashed line) VWs. (E-H) Morphometric measurements of transverse sections of the indicated genotype. (E) Tukey box-plot of the primary and secondary VW added together, forming the whole flank of the embryo. (F) Bar chart of the mean length of the primary (gray) and secondary (black) VW. (G) Tukey box-plot of the primary VW, showing no differences between any genotype. (H) Tukey box-plot of the secondary VW, showing a highly significant reduction in triple mutants. Box plots show median values (middle bars) and first to third interquartile ranges (boxes); whiskers indicate 1.5× the interquartile ranges; outliers in box-plots are plotted individually. Control *n=*8; TCre*;Fgf8^f/Δ^,17^Δ/Δ^,18^f/+^ n=*7; TCre*;Fgf8^f/+^,17^Δ/Δ^,18^f/Δ^ n=8*; triple mutant *n*=9. Significance was determined using a post-hoc Tukey-Kramer test. *P<0.05, **P<0.01, ***P<0.001, ****P<0.0001. Boxes indicate genotype, see Fig. 2 for key. Scale bars: 1 mm (A); 500 μm (C).

Having established that the window in which VW closure fails is between E12.5 and E13.5, we undertook a histological analysis and examined the primary VW and secondary VW at E13.5 and E12.5. Transverse sections through the abdomen at E13.5 showed that the secondary VW had migrated most of the way to the umbilical ring in both controls and triple mutants (Fig. 4A,B). We could also observe that in mutants the amniotic sac also contained a portion of the liver, and sometimes other organs too (Fig. 4B, red arrow). We measured the length of the embryonic flank from the dorsal muscle mass to the umbilical ring (Fig. 4E); the flank contains both the primary VW and secondary VW (Fig. 4F). All three mutant genotypes had significantly shorter flanks compared with controls, but triple mutants were the most severely affected. We measured the secondary VW and the primary VW individually (dashed yellow and red lines, respectively, in Fig. 4C,D). We found that although the length of the primary VW was unchanged (Fig. 4G), the primary VW was thinner in triple mutants (Fig. S6A-E). The secondary VW was much shorter in triple mutants when compared with any other genotype (Fig. 4H). We also saw that TCre*;Fgf8^f/Δ^;Fgf17^Δ/Δ^;Fgf18^f/+^* and TCre*;Fgf8^f/+^;Fgf17^Δ/Δ^;Fgf18^f/Δ^* embryos had a shorter secondary VW than controls, although in TCre*;Fgf8^f/+^;Fgf17^Δ/Δ^;Fgf18^f/Δ^* embryos this was just below significance (*P*=0.054).

**Fig. 5.**
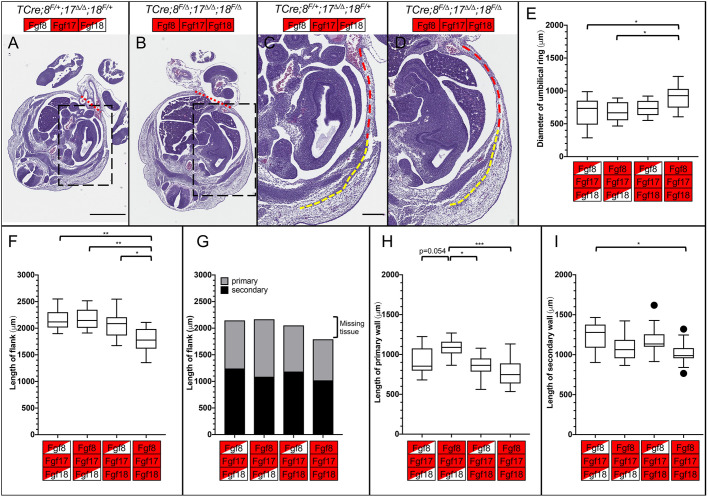
**Morphometric analysis of sections at E12.5 reveals changes in the primary and secondary VWs of *Fgf8* subfamily mutants preceding omphalocele.** (A,B) Transverse section of E12.5 control (A) and triple mutant (B) embryo at the interlimb region of the axis. Note the absence of liver in the physiological hernia of triple mutants at this stage. Red dashed lines indicate diameter of the umbilical ring, as measured in E. (C,D) Enlargement of boxed regions of A (C) and B (D) showing the primary (red dashed line) and secondary (yellow dashed line) VWs. (E-I) Morphometric measurements of transverse sections of the indicated genotype. (E) Tukey box-pot of the diameter of the umbilical ring. (F) Tukey box-plot of the primary and secondary VW added together, to form the whole flank of the embryo. (G) Bar chart of the mean length of the primary and secondary VW. The reduction in the length of the flank in triple mutants is indicated (bracket). (H) Tukey box-plot of the primary VW. (I) Tukey box-plot of the secondary VW. Box plots show median values (middle bars) and first to third interquartile ranges (boxes); whiskers indicate 1.5× the interquartile ranges; outliers in box-plots are plotted individually. Control *n=*12; TCre*;Fgf8^f/Δ^,17^Δ/Δ^,18^f/+^ n=*10; TCre*;Fgf8^f/+^,17^Δ/Δ^,18^f/Δ^ n=*11; triple mutant *n*=11. Significance was determined using a post-hoc Tukey-Kramer test. *P<0.05, **P<0.01, ***P<0.001. Boxes indicate genotype, see Fig. 2 for key. Scale bars: 1 mm (A); 250 μm (C).

We then performed the same analysis at E12.5, before omphalocele occurs in most mutants (Fig. 3D). We examined transverse sections and saw that the secondary VW had migrated about halfway to the umbilical ring in controls (Fig. 5A,C) and in triple mutants (Fig. 5B,D); we observed no liver in the physiological hernia in mutants. When we measured the diameter of the umbilical ring, we found that in triple mutants the umbilical ring was significantly wider (Fig. 5E), showing that an increase in umbilical ring diameter precedes the herniation of the liver and suggesting that this could cause the omphalocele. An enlarged umbilical ring presumably comes at the expense of the embryonic flank and, indeed, when we measured the flank we found that in triple mutants the flank was significantly shorter (Fig. 5F,G). We examined the primary VW and secondary VW to see if one was more affected than the other. In triple mutants the length of the primary VW was no different from controls, though it did trend shorter (Fig. 5H). The thickness of the primary VW was also unaffected at this stage in triple mutants (Fig. S6F-J), though the primary VW is thinner in TCre*;Fgf8^f/+^;Fgf17^Δ/Δ^;Fgf18^f/Δ^* embryos (Fig. S6I,J). The length of the secondary VW in triple mutants was significantly shorter than controls (Fig. 5I). TCre*;Fgf8^f/Δ^;Fgf17^Δ/Δ^;Fgf18^f/+^* embryos and TCre*;Fgf8^f/+^;Fgf17^Δ/Δ^;Fgf18^f/Δ^* embryos did not have a significantly shorter secondary VW, suggesting that one allele of *Fgf8* or *Fgf18* can rescue the length of the secondary VW at this embryonic stage. However, we did note that the TCre*;Fgf8^f/Δ^;Fgf17^Δ/Δ^;Fgf18^f/+^* embryos trended towards a shorter secondary VW, suggesting that the secondary VW was beginning to be affected at E12.5 (Fig. 5I). The trend towards a longer primary VW in TCre*;Fgf8^f/Δ^;Fgf17^Δ/Δ^;Fgf18^f/+^* embryos compared with controls (Fig. 5H) would explain why the total flank in TCre*;Fgf8^f/Δ^;Fgf17^Δ/Δ^;Fgf18^f/+^* embryos is unchanged (Fig. 5F,G). We also examined proliferation and cell death at E12.5. Anti-pHH3 and activated c-caspase 3 co-immunostaining at E12.5 in the secondary VW of the embryo (Fig. S7A-D) did not reveal any changes between genotypes. This suggests that the reduction in secondary VW length is primarily due to a defect in migration from the somites, or because the somites were formed with less tissue before muscle formation.

In triple mutants the entire flank, which includes the primary and secondary VWs, was shorter than controls at E12.5 (Fig. 5F). We propose that in triple mutants these VW defects result in an enlarged umbilical ring and therefore a higher rate of omphalocele. At E13.5, all three mutant genotypes have a shorter flank than controls (Fig. 4E), and this reduction in length is largely the result of a shorter secondary VW (Fig. 4H). This reduction in length is most pronounced in triple mutants. From these morphometric analyses we conclude that the VW defects we observe start earlier in triple mutants and are more severe than in TCre*;Fgf8^f/Δ^;Fgf17^Δ/Δ^;Fgf18^f/+^* embryos and TCre*;Fgf8^f/+^;Fgf17^Δ/Δ^;Fgf18^f/Δ^* embryos, strongly suggesting an additive genetic effect.

### *Fgf8* is required in the PSM and *Fgf18* is required in the somites to close the VW

Although we have been able to show that the *Fgf8* subfamily is needed for VW closure, we did not know when and where these genes are required. TCre recombination begins at E7.5 in the primitive streak and nascent PSM (Perantoni et al., 2005). Therefore, the structures that close the VW, the LPM-derived primary VW and the somite-derived abdominal muscles, will both contain deleted *Fgf* alleles. Although the expression of *Fgf17* is limited to the PSM, *Fgf8* and *Fgf18* are expressed in both the PSM and the somites, making it impossible to determine their spatial and temporal requirements using TCre. We decided to use two other Cre-expressing lines (see Table S1), the *Meox1^tm1(cre)Jpa^* (hereafter ‘Meox1Cre’) line, which recombines in the somites after segmentation (Jukkola et al., 2005), and the Tg(Cited1-cre/ERT2,-EGFP)1Mdca or Cited1CreER^T2^ (hereafter ‘Cited1Cre’) line, which recombines in the PSM and somites but not the primitive streak (Boyle et al., 2008; Garriock et al., 2015).

**Fig. 6.**
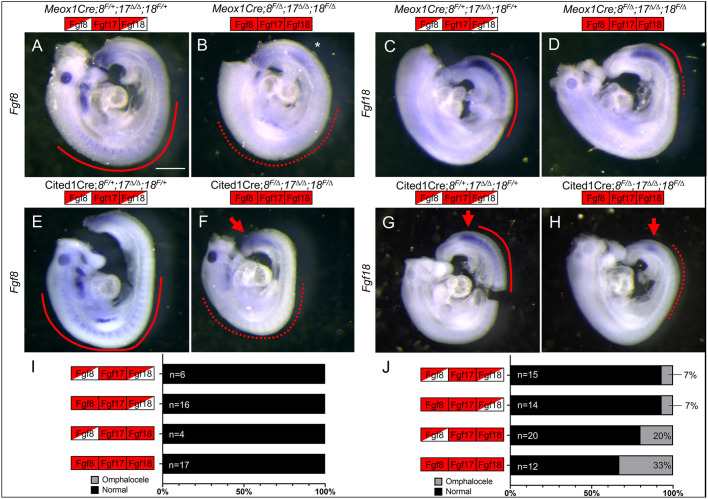
**Different tissue-specific Cre lines demonstrate requirement for *Fgf8* in PSM and *Fgf18* in somites.** (A-D) WISH directed against floxed region of *Fgf8* (A,B) and *Fgf18* (C,D) in Meox1Cre*;Fgf8^f/+^;Fgf17^Δ/Δ^;Fgf18^f/+^* embryos (A,C) and Meox1Cre*;Fgf8^f/Δ^;Fgf17^Δ/Δ^;Fgf18^f/Δ^* embryos (B,D). Meox1Cre*;Fgf8^f/+^;Fgf17^Δ/Δ^;Fgf18^f/+^* embryos displayed normal expression of *Fgf8* (A) and *Fgf18* (C) in the somites (red line). Meox1Cre*;Fgf8^f/Δ^;Fgf17^Δ/Δ^;Fgf18^f/Δ^* embryos lost expression of *Fgf8* (B) in the somites (red dashed line), but *Fgf18* was mostly intact (D). Ectopic expression of *Fgf8* in Meox1Cre embryos (A,B), indicated by asterisk, compare with (E). (E-H) WISH directed against floxed region of *Fgf8* (E,F) and *Fgf18* (G,H) in Cited1Cre*;Fgf8^f/+^;Fgf17^Δ/Δ^;Fgf18^f/+^* embryos (E,G) and Cited1Cre*;Fgf8^f/Δ^;Fgf17^Δ/Δ^;Fgf18^f/Δ^* embryos (F,H). Cited1Cre*;Fgf8^f/Δ^;Fgf17^Δ/Δ^;Fgf18^f/Δ^* embryos lost expression of *Fgf8* (F) and *Fgf18* (H) in the somites (red dashed line). Note that expression of *Fgf8* in the PSM is unaffected in mutants (F, red arrow), and the expression of *Fgf18* is still present in the anterior PSM (H, red arrow). (I,J) Graphical representation of the incidence of omphalocele at E18.5 using Meox1Cre (I) and at E15.5 using Cited1Cre (J) showing the percentage of embryos with and without omphalocele. The total number of embryos is to the left of the bars and the percentage of embryos with omphalocele is on the right. Boxes indicate genotype, see Fig. 2 for key. Scale bar: 500 μm.

We used the *Gt(ROSA)26Sor^tm1Sor^*/J (hereafter ‘R26R’) reporter line (Soriano, 1999) to examine the Meox1Cre recombination pattern. Recombination in the most posterior somites was incomplete; strong signal was only observed about four somites anterior to the PSM (Fig. S8A). We performed WISH against the floxed region of *Fgf8* (Fig. 6A,B) and *Fgf18* (Fig. 6C,D) to confirm that Cre recombination had occurred. In *Meox1Cre;Fgf8^f/Δ^;Fgf17^Δ/Δ^;Fgf18^f/Δ^* embryos we saw strong *Fgf8* expression in the PSM but no expression in the somites, as expected. We also observed an ectopic region of *Fgf8* expression in several caudal somites that we have determined is a consequence of losing a copy of *Meox1*, as Cre is inserted into the *Meox1* locus (Jukkola et al., 2005). In *Meox1Cre;Fgf8^f/Δ^;Fgf17^Δ/Δ^;Fgf18^f/Δ^* embryos we saw a restriction in the anterior extent of the somitic *Fgf18* expression and a slight decrease in transcript levels in the PSM and somites. *Meox1Cre;Fgf8^f/Δ^;Fgf17^Δ/Δ^;Fgf18^f/Δ^* embryos never presented with omphalocele at E18.5 (Fig. 6I, Fig. S8B). As recombination is complete in the dermomyotome by E9.5 (Jukkola et al., 2005), the downstream abdominal muscles will have been recombined, so there must be no later requirement in VW closure for *Fgf8* and *Fgf18* in this tissue lineage.

We next used the Cited1Cre line to recombine the *Fgf8* subfamily after tamoxifen induction (see Materials and Methods). We examined the recombination pattern in Cited1Cre*;R26R* embryos at E9.5 and saw complete recombination in the anterior PSM, the LPM and the somites throughout the axis, although the occipital somites remained unrecombined (Fig. S8C-G). We confirmed that robust recombination was observed caudal to the hindlimbs in E11.5 embryos (Fig. S8H), thus controlling gene expression in the somites that will give rise to the abdominal secondary VW. We checked that floxed alleles were recombined using WISH. *Fgf8* transcripts remained in the PSM, but not the somites, in *Cited1Cre;Fgf8^f/+^;Fgf17^Δ/Δ^;Fgf18^f/Δ^* embryos (Fig. 6E,F). This is because the gradient of *Fgf8* mRNA in the PSM is a result of long-lived transcripts made in PSM progenitors in the caudal-most tailbud (Fig. S8I), outside the Cited1Cre expression domain (Dubrulle and Pourquié, 2004). *Fgf18* expression in the PSM was reduced and expression in the somites was abolished in Cited1Cre*;Fgf8^f/+^;Fgf17^Δ/Δ^;Fgf18^f/Δ^* embryos (Fig. 6G,H).

We then scored omphalocele in embryos with Cited1Cre-mediated deletion of the *Fgf8* subfamily. Spontaneous abortions due to tamoxifen administration prevented recovery of E18.5 embryos often enough to impede our efforts. However, we reliably recovered E15.5 embryos at Mendelian ratios, which allowed analysis because the phenotype was evident by E13.5 (Fig. 3). In Cited1Cre*;Fgf8^f/Δ^;Fgf17^Δ/Δ^;Fgf18^f/+^* embryos, the omphalocele incidence was very infrequent and equivalent to controls (one individual in each genotype) (Fig. 6J), suggesting that the loss of *Fgf8* expression in the somites (Fig. 6F) does not cause a VW closure defect and therefore the *Fgf8* requirement is in the PSM. In Cited1Cre*;Fgf8^f/+^;Fgf17^Δ/Δ^;Fgf18^f/Δ^* embryos, the omphalocele frequency was similar to that observed in TCre*;Fgf8^f/+^;Fgf17^Δ/Δ^;Fgf18^f/Δ^* embryos (Fig. 2G), suggesting the requirement for *Fgf18* in VW closure is in the somites. The lack of a phenotype in *Meox1Cre*;*Fgf8^f/Δ^;Fgf17^Δ/Δ^;Fgf18^f/Δ^* embryos is a result of two intact activities: *Fgf8* in the PSM and *Fgf18* in the somites (Fig. 6A-D). Cited1Cre*;Fgf8^f/Δ^;Fgf17^Δ/Δ^;Fgf18^f/Δ^* embryos displayed a 33% rate of omphalocele (Fig. 6J, Fig. S8J), which was significantly lower than the 71% observed in TCre*;Fgf8^f/Δ^;Fgf17^Δ/Δ^;Fgf18^f/Δ^* embryos (*P*=0.0139, two-tailed Fisher's exact test) because in the Cited1Cre*;Fgf8^f/Δ^;Fgf17^Δ/Δ^;Fgf18^f/Δ^* embryos the PSM domain of *Fgf8* remained intact (Fig. 6F).

From these results we conclude that the requirement for *Fgf8* expression is in the PSM; the hypothesis that *Fgf8* is required in the PSM is further supported by the fact that loss of *Fgf17* in TCre*;Fgf8^f/Δ^* mutants promoted omphalocele (Fig. 2G), and *Fgf17* is expressed in the PSM but not the somites (Fig. 1). The requirement for the expression of *Fgf18* is in the anterior PSM and/or newly formed somites. The lack of a VW defect in *Meox1Cre;Fgf8^f/Δ^;Fgf17^Δ/Δ^;Fgf18^f/Δ^* embryos (Fig. 6I) suggests there is no requirement in the dermomyotome or later myotome or muscle lineages for *Fgf8* or *Fgf18*.

### The *Fgf8* subfamily is required to maintain proper levels of progenitor tissue in the PSM and correctly sized somites

**Fig. 7.**
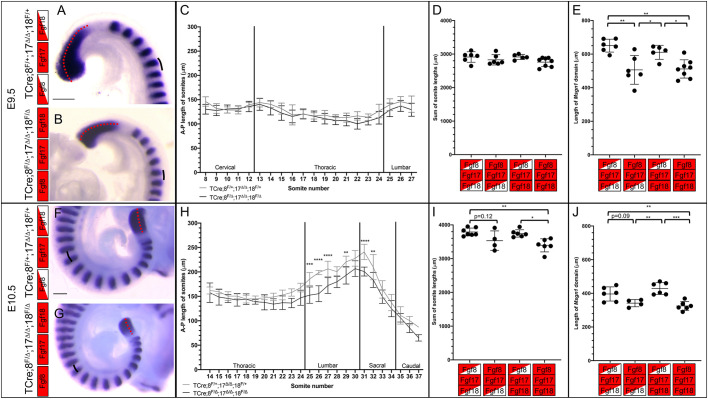
**Fgf8 is required to maintain the length of the *Msgn1* domain and the size of the somites.** (A,B) Double WISH for *Msgn1* and *Uncx4.1* on E9.5 controls (A) and triple mutants (B). The length of the PSM (red dotted line) and the A-P length of the somites (black line indicates a representative example) were measured. (C) Graph of the A-P length of each somite for controls (gray line and bars, *n=*6) and triple mutants (black line and bars, *n*=8) at E9.5. Data are mean±s.d.; multiple *t*-test with Holm–Šidák correction applied. (D) Total A-P length of somites 12-24 at E9.5. (E) Length of the *Msgn1* domain. (F,G) Double WISH for *Msgn1* and *Uncx4.1* on E10.5 controls (F) and triple mutants (G). (H) Graph of the A-P length of each somite for controls (gray line and bars, *n*=7) and triple mutants (black line and bars, *n*=6) at E10.5. Data are mean±s.d., multiple *t*-test with Holm-Šidák correction applied. (I) The total A-P length of somites 14-35 at E10.5. (J) Length of *Msgn1* domain at E10.5. In panels D, E, I and J, data are individually plotted, mean and s.d. are shown, a post-hoc Tukey-Kramer test applied. **P<0.01, ***P<0.001, ****P<0.0001. Boxes indicate genotype, see Fig. 2 for key. Scale bars: 250 μm.

The results we have obtained thus far present something of a conundrum: how does the loss of *Fgf8* and *Fgf17* in the PSM cause omphalocele at E13.5 and later? As FGF signaling is known to be essential for both maintaining a pool of progenitor tissue within the PSM (Naiche et al., 2011) and for somitogenesis (Dubrulle et al., 2001), we looked for defects in these two processes in our TCre *Fgf8* subfamily conditional mutant mouse line. We chose the TCre line because triple mutants have the highest incidence of omphalocele, so we can analyze embryos that would likely have had omphalocele at a later gestational age. We performed WISH for both *Msgn1* and *Uncx4.1* (*Uncx*) at E9.5 (Fig. 7A,B) and at E10.5 (Fig. 7F,G), to label the progenitors of the paraxial mesoderm (Yoon et al., 2000) and the caudal half of each somite, respectively (Mansouri et al., 1997), in somite-stage matched E9.5 and E10.5 embryos.

Using *Uncx4.1* expression*,* at the 28-30 somite stage (E9.5) we could see a trend towards a smaller anterior-posterior (A-P) length in each somite throughout the length of triple mutants (Fig. 7C). We calculated the sum of the lengths of somites 12-27 (corresponding to the future thoracic and lumbar vertebrae) and found no differences between triple mutants and controls (Fig. 7D). We then examined the PSM, as a defect in the PSM could lead to somite defects. We observed that the *Msgn1* expression domain was shorter in triple mutants compared with controls (Fig. 7E). We also observed a shorter *Msgn1* expression domain in TCre*;Fgf8^f/Δ^;Fgf17^Δ/Δ^;Fgf18^f/+^* but not TCre*;Fgf8^f/+^;Fgf17^Δ/Δ^;Fgf18^f/Δ^* embryos compared with controls (Fig. 7E).

We then examined the expression of *Msgn1* and *Uncx4.1* approximately 24 h later (E10.5), at the 35-37 somite stage (Fig. 7F,G). We saw a far greater reduction in the A-P length of the somites of triple mutants compared with controls, with the length of multiple individual somites in the lower thoracic, lumbar and sacral region of the axis being smaller (Fig. 7H). There was a significant reduction in the sum of the A-P length of somites 14 through 35, which give rise to the vertebrae and muscles of the thorax and abdomen, in triple mutants compared with controls (Fig. 7I). As was the case at E9.5, there was a significant reduction in the length of the *Msgn1* domain in E10.5 triple mutants compared with controls (Fig. 7J). As was observed at E9.5, a reduction in the *Msgn1* domain was evident in TCre*;Fgf8^f/Δ^;Fgf17^Δ/Δ^;Fgf18^f/+^* but not TCre*;Fgf8^f/+^;Fgf17^Δ/Δ^;Fgf18^f/Δ^* embryos.

**Fig. 8.**
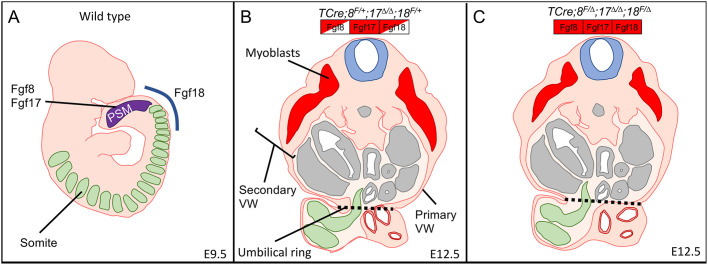
**Diagrams linking the spatial requirements of the *Fgf8* subfamily to VW morphogenesis.** (A) Diagram of a wild-type E9.5 embryo showing *Fgf8* and *Fgf17* requirement in the PSM (purple) and *Fgf18* requirement in the anterior PSM (blue line). (B) Diagram of an E12.5 embryonic transverse section with the primary VW, secondary VW and umbilical ring (dotted line) indicated. (C) Triple mutant E12.5 embryonic transverse section showing smaller secondary VW and enlarged umbilical ring (dotted line).

Together, the results of this paper show that *Fgf8*, but not *Fgf18*, is required for maintaining proper length of the PSM (as indicated by the *Msgn1* domain) and that in the absence of the *Fgf8* subfamily the size of the lumbar and sacral somites is reduced. The loss of *Fgf17*, which is expressed only in the PSM, likely contributes to this reduction of the PSM progenitor domain. Our genetic analysis shows that *Fgf8* and *Fgf17* are required in the PSM and that *Fgf18* is required in the somites (Fig. 8A). In controls, the migration of the secondary VW and size of the umbilical ring is normal (Fig. 8B), but in triple mutants the secondary VW is shorter and the umbilical ring is larger (Fig. 8C), which leads to omphalocele.

## DISCUSSION

Inactivating multiple genes often reveals the complexity that underlies genetic interactions during embryogenesis. The 18 signaling members of the FGF ligand family frequently play numerous essential and redundant roles in development (reviewed by Ornitz and Itoh, 2015). Such interactions occur across subfamilies, such as the redundancy between *Fgf4* and *Fgf8* in maintenance of the undifferentiated PSM (Naiche et al., 2011) and in limb bud outgrowth (Boulet et al., 2004), *Fgf9* and *Fgf18* in skeletal development (Hung et al., 2016), or they occur within a subfamily, such as between *Fgf8* and *Fgf17* in the hindbrain (Xu et al., 2000), *Fgf3* and *Fgf10* in otic placode and cardiovascular development (Urness et al., 2011; Wright and Mansour, 2003), *Fgf3* and *Fgf8* in otic placode development (Ladher et al., 2005), and *Fgf9* and *Fgf20* in cochlea, tooth and kidney development (Barak et al., 2012; Haara et al., 2012; Huh et al., 2015; Yang et al., 2019).

We present here the first evidence for genetic redundancy between all three members of the *Fgf8* subfamily. By inactivating these Fgf genes in different embryonic subsets using different tissue-specific Cre lines, we found that the activities of FGF8 and FGF17 are required in the PSM, and that FGF18 is required in the somites, in order to close the VW. By measuring somite and PSM progenitor domain length, we found that *Fgf8* and *Fgf17* appear to play a role in maintaining proper somite size. We propose that smaller somites cause a reduction in the amount of tissue in the secondary VW, and our morphometric analysis suggests that a loss of FGF18 activity can impair the secondary VW by delaying muscle migration. Defects in either somite size or muscle migration results in a low incidence of omphalocele, but when they occur simultaneously, the omphalocele incidence is greatly increased. This phenomenon, where defects in two separate processes result in a phenotype, is reminiscent of the *Fgf3;Fgf4* double mutant. In this mouse line a loss of *Fgf3* results in a reduction in levels of *Fgf8* (Anderson et al., 2016a) and as this is on an *Fgf4* mutant background, the FGF signal that maintains the PSM in an undifferentiated state is lost (Naiche et al., 2011). Consequently, the loss of tail vertebrae is more severe in the *Fgf3;Fgf4* double mutant than in *Fgf3* nulls alone (*Fgf4* mutants have a normal length tail) (Anderson et al., 2016b).

Furthermore, this study is the first to describe a role for FGF ligands in VW closure, and we demonstrate a gene dosage effect on the incidence of omphalocele. Omphalocele is also observed in *Fgfr1* heterozygotes, when *Fgfr2* is also conditionally inactivated at E8.5 throughout the embryo (Nichol et al., 2011). *Fgfr1* transcripts are detected in the PSM and both *Fgfr1* and *Fgfr2* genes are expressed in the somites and LPM at E8.5 and E9.5. (Orr-Urtreger et al., 1991; Wahl et al., 2007). Thus, the expression domains of these receptor genes are consistent with our model (Fig. 8). At E11.5-E13.5, these receptor genes are also expressed in the VW itself; *Fgfr1* in a broad domain and *Fgfr2* transcripts are detected in VW subsets (Nichol et al., 2011; Orr-Urtreger et al., 1991; Peters et al., 1992). These later expression domains of the *Fgfr* genes suggest that FGF signaling may act at multiple locations to close the VW, a complexity similar to the role of FGF signaling in limb development (Benazet and Zeller, 2009; Jin et al., 2018). If FGFs provide directional cues for secondary VW migration at these later stages, as they do during migration of tracheal cartilage (Elluru et al., 2009), our data suggest they may be encoded by Fgfs outside the *Fgf8* subfamily. This later FGF signaling step may be upstream of MEK1 and MEK2 function, which is required at these stages to close the VW (Boucherat et al., 2014). Future work will determine whether MEK1/2 kinases function downstream of the FGF8 subfamily in the PSM and somites.

In humans, omphalocele is reported in two case studies of Aperts syndrome (Ercoli et al., 2014; Herman and Siegel, 2010), which is caused by Fgfr2 mutations, but is otherwise known as a craniosynostosis pathology (Armand et al., 2019). Otherwise, there are no reports of mutations in Fgf ligands or receptors causing VW defects in humans, possibly because of genetic redundancy or embryonic lethality.

Kyphosis has been proposed as a causal factor in several mouse models of omphalocele (Boucherat et al., 2014; Kakizaki et al., 2015) with the rationale that a malpositioned spine could reduce the volume of the abdominal cavity and increase intra-abdominal pressure, forcing the viscera out through the umbilicus. We observed that triple mutants, compared with *TCre^+^;Fgf8^f/+^;Fgf17^Δ/Δ^;Fgf18^f/Δ^* embryos, have both a greater rate of omphalocele and more severe kyphosis, suggesting spinal defects may exacerbate VW defects. However, it is not the case that kyphosis is the primary cause, because omphalocele occurs in *TCre^+^;Fgf8^f/+^;Fgf17^Δ/Δ^;Fgf18^f/+^* mutants, which lack kyphosis, demonstrating that VW defects occur with a normal spine. Furthermore, in human patients, once the omphalocele is repaired, the volume of the abdominal cavity and the posture of the spine both recover (Nagaya et al., 2000), suggesting omphalocele may cause spine defects. Therefore, any causality between kyphosis and omphalocele is unclear and warrants future study.

We see a measurable delay in mutant muscle migration at E12.5 and E13.5, and it has been proposed that muscle migration defects of the secondary VW are causative of omphalocele (Nichol et al., 2012). Defects in the primary VW have also been linked to VW closure defects (Brewer and Williams, 2004b), and it is known that the primary VW acts as a signaling center to promote secondary VW morphogenesis (Aldeiri et al., 2017; Brewer and Williams, 2004b; Nichol et al., 2011; Zhang et al., 2014). In triple mutants at E12.5 the whole flank is smaller and the umbilical ring is larger immediately before omphalocele. We speculate that as the embryo grows the viscera are forced through this enlarged opening.

Using multiple Cre lines, we determined the spatial and temporal requirements for the Fgf8 subfamily genes in VW closure. Consistent with the hypothesis that omphalocele is multifactorial in origin, we find that *Fgf8* and *Fgf17* are needed in the PSM whereas *Fgf18* is required in the somites. This requirement for *Fgf8* and *Fgf17* in the PSM is supported by the observation that in TCre*^+^;Fgf8^f/Δ^;Fgf17^Δ/Δ^;Fgf18^f/+^* mutants, there is a reduction in the PSM *Msgn1* domain, which marks the progenitor pool of cells that form the mesoderm, including the LPM and the paraxial mesoderm. As a result, the somites are smaller and there is less material available to close the VW. This, combined with the secondary VW defects or the kyphosis caused by the loss of *Fgf18*, causes the high rates of omphalocele that we observe in triple mutants. In addition, we suggest that FGF signals do not provide directional cues per se for the secondary VW lineage, but are generally required for migration, as is the case for FGF signaling in limb and axis extension (Benazeraf et al., 2010; Gros et al., 2010; Lewandoski and Mackem, 2011). This idea is the focus of our future work. Our data highlight the complex and multifactorial origins of omphalocele and demonstrate that one simple overarching model for explaining its etiology is likely to be insufficient.

## MATERIALS AND METHODS

### Animal husbandry

Animals were maintained in accordance with the recommendations in the Guide for Care and Use of Laboratory Animals of the National Institutes of Health under a protocol approved by the Animal Care and Usage Committee of NCI at Frederick (NIH) (Animal Study Proposal: 17-069). Mice of both sexes were utilized, and all lines were maintained on an outbred background.

To conditionally delete *Fgf8* and *Fgf18* activity we used the previously described *Fgf8^flox^* line (Meyers et al., 1998) (hereafter *Fgf8^f^*) and the *Fgf18^flox^* line (Hagan et al., 2019) (hereafter *Fgf18^f^*). Null alleles of these genes as well as *Fgf17* were generated as previously described (Hagan et al., 2019; Meyers et al., 1998; Xu et al., 2000).

The TCre, Meox1Cre and Cited1Cre lines have all been described previously (Boyle et al., 2008; Jukkola et al., 2005; Perantoni et al., 2005). In order to generate experimental crosses, females homozygous for floxed alleles were mated to Cre recombinase-positive males. The same line of females was used for TCre, Meox1Cre and Cited1Cre experiments in which mutants for all three members of the *Fgf8* subfamily were generated. We used the *Gt(ROSA)26Sor^tm1Sor^*/J (R26R) (Soriano, 1999) and *Gt(ROSA)26Sor^tm4(ACTB-tdTomato,-EGFP)Luo^*/J (*mTmG*) lines (Muzumdar et al., 2007) to reveal Cre recombined tissues.

### Tamoxifen administration

For embryonic experiments tamoxifen (40 mg/kg) and progesterone (40 mg/kg) were co-injected intraperitoneally into the same pregnant dams at 10:00 AM on the morning of E7.5 and E8.5; embryos were collected at E15.5. This ensured that all mesoderm from the level of the heart until several somites caudal to the hindlimbs (i.e. past the sacral vertebrae) was recombined. To prepare, 100 mg of tamoxifen (Millipore-Sigma, T5648) was dissolved in 5 ml of corn oil (Millipore-Sigma, C8267) to which was added 5 ml of 50 mg/ml progesterone dissolved in sesame oil (Watson Pharma Inc., NDC 0591-3128-79). This was then sterilized through a 0.22 µm filter and stored at 4°C before use.

### Wish

WISH was performed according to published protocols (Wilkinson and Nieto, 1993). In order to bring up regions of the embryo with relatively low levels of expression, development of the stain was prolonged. For the recombination probe experiment, controls and mutants were processed in the same vial and developed for the same length of time at 37°C. The *Fgf8* and especially the *Fgf18* recombination probes took considerable time to develop. We noticed that fainter domains of *Fgf18* were never labeled with the *Fgf18* recombination probe, presumably because the signal-to-noise ratio was too poor in these regions. Samples from E9.5 to E11.5 were cleared in 50% glycerol:PBS for several days before imaging. The *Fgf8*, *Fgf17* and *Fgf18* full-length probes have been previously reported (Maruoka et al., 1998), as has the *Fgf8* recombination probe (Perantoni et al., 2005). The PCR primers to generate the template for the *Fgf18* recombination probe (reverse primer also includes a T7 RNA polymerase promotor sequence) are: forwards, AGCCGAGGAGAATGTGGACT; reverse, TAATACGACTCACTATAGGGCCCAGGACTTGAATGTGCTT.

### Paraffin sections

For morphometric analyses of E12.5 and E13.5 embryos, samples were fixed overnight or longer in 10% neutral buffered formalin. They were taken through a graded series of ethanol dilutions before being infiltrated with xylene and then paraffin wax in a vacuum chamber. Then, 8 µm sections were taken and stained with Eosin and Hematoxylin (E12.5) or Masson's trichrome stain (E13.5). Stained slides were imaged and morphometric data obtained using Fiji image analysis software (Schindelin et al., 2012). For E18.5 embryos, samples were decapitated and the heart and lungs removed from the thoracic cavity before the whole sample was immersed in Bouin's solution for at least 7 days. Samples were then processed as for younger stages and stained with Masson's trichrome.

### Morphometric analysis

A representative section for each stage-matched embryo was selected and all measurements were taken using that section. Sections were taken from the same axial level of the embryo and positionally matched using the stomach, kidneys, liver and gonads as anatomical markers.

### Skeletal preparations

Alcian blue and Alizarin red co-staining was performed as previously described (Nagy et al., 2003).

### Statistical analyses

When testing for significant differences in the rate of omphalocele between different genotypes or between different timepoints of the same genotype, a two-tailed Fisher's exact test was used. This test was also used when looking at the yolk sac defects at E10.5. When determining Mendelian ratios, a Chi-squared test was used. To perform multiple comparisons of the morphometric measurements of embryonic sections at E12.5 and E13.5, a post-hoc Tukey–Kramer test was used. This test was also used for the cell division and cell death analyses. In order to find whether there were differences between specific somites, a multiple *t*-test with a Holm-Šidák correction was used to account for type 1 errors (Holm, 1979). When investigating the total A-P length of the somites, and for investigating the length of the *Msgn1* domain, a post-hoc Tukey–Kramer test was used.

### Antibody staining

Samples were fixed overnight at 4°C in 4% paraformaldehyde, then dehydrated and infiltrated with paraffin by hand. After sectioning, samples were dewaxed, subjected to antigen retrieval and stained according to Anderson et al. (2016a) with the following modifications: Citrisolv (Thermo Fisher Scientific) was used for dewaxing, sodium citrate buffer containing 0.05% Tween 20 (Sigma-Aldrich) and 10% normal goat serum (Sigma-Aldrich) in PTX was used for dilution of blocking and antibodies. Primary antibodies used were anti-phospho histone H3 (Cell Signaling Technologies, 9706L, 1:500) and anti-cleaved caspase 3 (Cell Signaling Technologies, 9661L, 1:250). Secondary antibodies used were goat anti-mouse Alexa Fluor 594 (Thermo Fisher Scientific, A-32740) and goat anti-rabbit Alexa Fluor 647 (Thermo Fisher Scientific, A-21244). Immunocomplexes were detected using a Zeiss LSM710 confocal microscope using a 20× objective to generate tiled *z*-stacks. Nuclei were counted using Imaris image analysis software (Oxford instruments). pHH3- and cleaved caspase 3-positive cells were counted manually using maximum projections of *z*-stacks. Three sections per embryo were analyzed to generate results in triplicate.

### HCR

HCR was performed as previously described (Choi et al., 2018) with modifications as described in Anderson et al. (2020 preprint). Probes against *Fgf8*, *Fgf17* and *Fgf18* were designed by Molecular Instruments and were hybridized with hairpins conjugated to Alexa Fluor 647, Alexa Fluor 546 and Alexa Fluor 488 (Molecular Instruments), respectively. After hybridization, samples were embedded in ultra low melt agarose on a glass bottomed dish and cleared in Ce3D as previously described (Anderson et al., 2020 preprint; Li et al., 2017). After clearing, samples were imaged on a Nikon A1 confocal microscope and captured *z*-stacks were processed in FiJi (Schindelin et al., 2012).

### β-galactosidase staining

Pregnant dams were euthanized and embryos were dissected out as previously described. Embryos were then fixed for 40 min at room temperature in fixation buffer (1% formaldehyde, 0.2% glutaraldehyde, 2 mM MgCl_2_, 5 mM EGTA and 0.02% NP-40 in PBS). Embryos were then washed 3×10 min in 0.02% NP-40 in PBS solution. Embryos were then incubated at 37°C overnight in stain solution (5 mM potassium ferricyanide, 5 mM potassium ferrocyanide, 2 mM MgCl_2_, 0.01% sodium deoxycholate, 0.02% NP-40, 1 mg/ml X-gal in PBS), After staining was complete, embryos were washed in PBS and then post fixed in 4% paraformaldehyde at 4°C overnight. For whole-mount imaging embryos were then washed in PBS, cleared in 50% glycerol and imaged. Embryos for sectioning were put through a dehydration ethanol series, embedded in paraffin and 8 µm sections were taken on a microtome (Lecia Reichert Jung BioCut 2030). Slides were then dewaxed, stained with Nuclear Fast Red and then coverslipped and imaged.

### Genotyping primers

Fgf18 flox: forwards, ATTCAGGAGCAGCTCAGTCC; reverse, TGTCATGACCTGATGGCAAC. Fgf18 delta: forwards, CCTGGGGCTGTGGGAAAATA; reverse, GCCTGGGGTTGATGTGTACT. TCre: forwards, GCTGTTGGGTAGGGAGTCAA; reverse, ATGTTTAGCTGGCCCAAATG.

## Supplementary Material

Supplementary information

Reviewer comments
