# Supplementary material for: The Fgf8 subfamily (Fgf8, Fgf17 and Fgf18) is required for closure of the embryonic ventral body wall
Source: Development. 2020 Oct 19;147(21):dev189506. doi: 10.1242/dev.189506 (PMC7595690; doi:10.1242/dev.189506)
Supplement: Supplementary information [file develop-147-189506-s1.pdf]

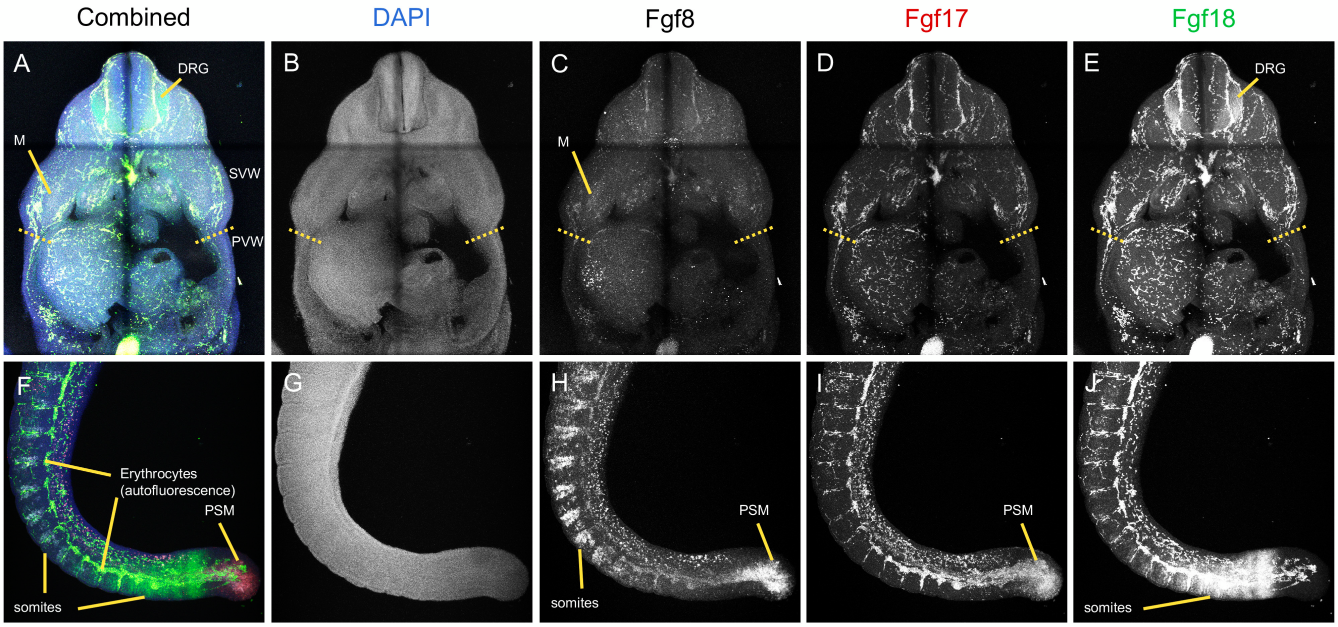

Figure S1. Expression of the *Fgf8* subfamily genes is undetectable in the E11.5 ventral body wall. Max intensity projection (MIP) of a Z-stack of E11.5 wildtype embryos stained for expression of *Fgf8*, *Fgf17* and *Fgf18* by hybridization chain reaction (Choi et al., 2018) . (A-E) Transverse image showing staining of DAPI (B), *Fgf8* (C), *Fgf17* (D), *Fgf18* (E). Note weak expression of *Fgf8* in the myotome (M) (A, C) and of *Fgf18* in the dorsal root ganglion (DRG) (A, E). There is no detectable expression of the Fgf8 subfamily genes in the secondary or primary ventral walls (separated by yellow dotted line in A–E). Lateral image of E11.5 tail showing previously established expression domains for *Fgf8* (H) *Fgf17* (I) and *Fgf18* (J) in somites and PSM. Note high autofluorescence of erythrocytes in all channels.

Development • Supplementary information

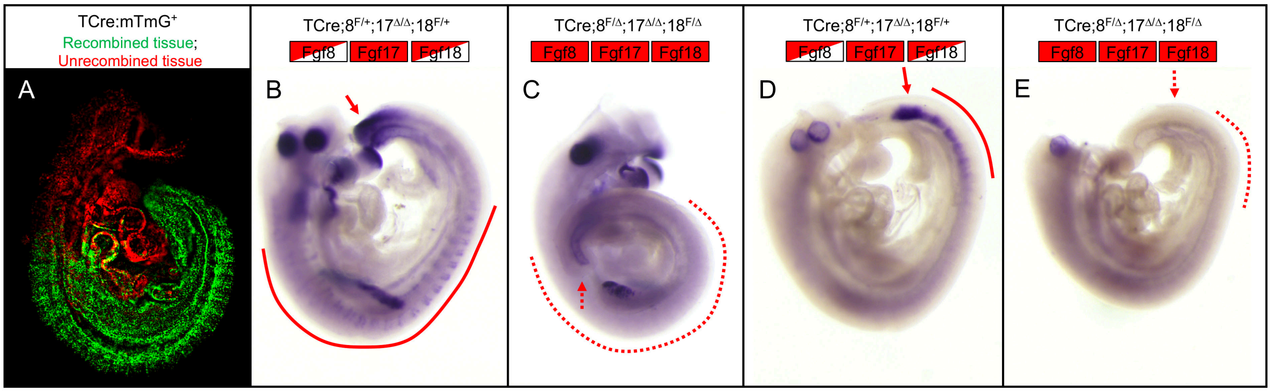

Figure S2. Characterization of TCre-mediated deletion of *Fgf8* and *Fgf18* (A) TCre; *mTmG* E9.5 embryo showing complete recombination in the PSM, the somites and LPM (green). (B,C) WISH using probe directed against the recombined region of *Fgf8* in E9.5 embryos. *Fgf8* transcript is detected as expected in the control embryo (B, arrow, PSM and line, somites) and is missing in the mutant embryo (C, dashed arrow and line). (D,E) WISH using probe directed against the recombined region of *Fgf18* in E9.5 embryos. *Fgf18* transcript is present as expected in control embryo (D, arrow, anterior PSM and line, somites) and is missing in the triple mutant embryo (E, dashed arrow and line). Boxes indicate genotype, see Fig. 2 for key.

Development • Supplementary information

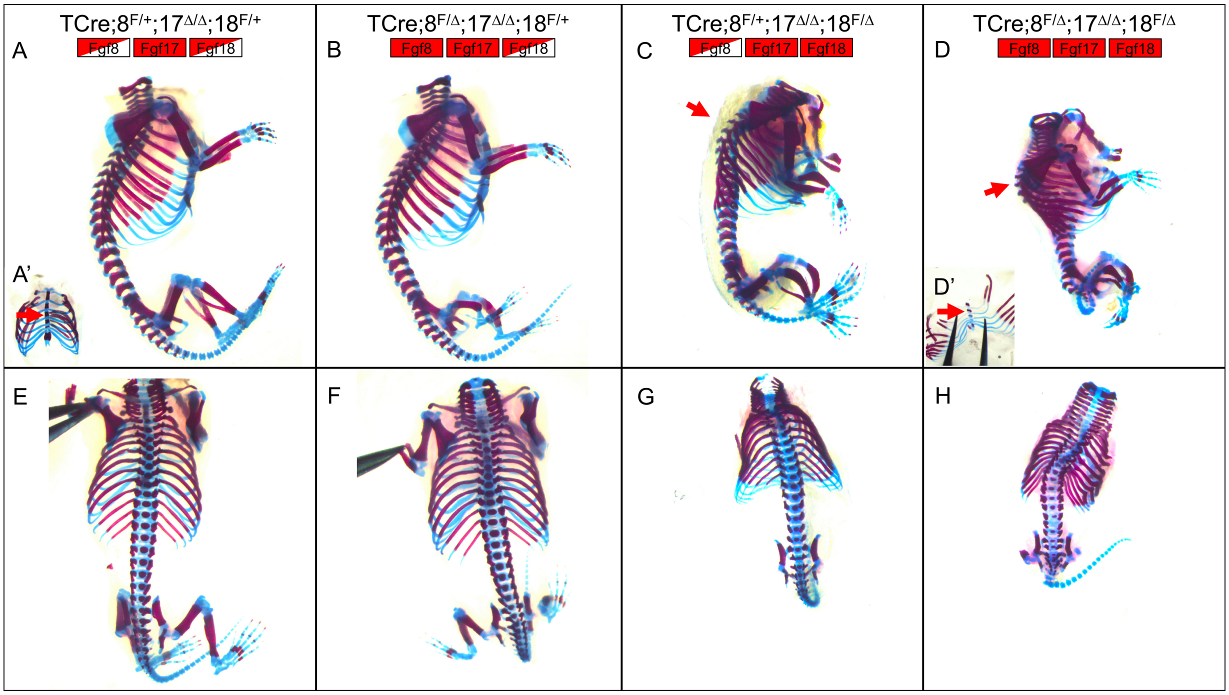

Figure S3. Loss of *Fgf8* and *Fgf17* exacerbates the skeletal defects seen in *Fgf18* loss-of-function embryos embryos at E18.5. (A-H) Alcian blue and alizarin red staining of skeletons of control (A,E), TCre;*Fgf8*$^{f/\Delta}$*,17*$^{\Delta/\Delta}$*,18*$^{f/+}$ (B,F); TCre;*Fgf8*$^{f/+}$*,17*$^{\Delta/\Delta}$*,18*$^{f/\Delta}$ (C,G) and triple mutant (D,H) embryos. Inserts A' and D' are ventral views of the indicated genotype showing correct sternum fusion (red arrows). E,F,G and H are a dorsal view of the same embryo in A,B,C and D, respectively. Note the kyphosis in C and D (arrow). Boxes indicate genotype, see Fig. 2 for key.

Development • Supplementary information

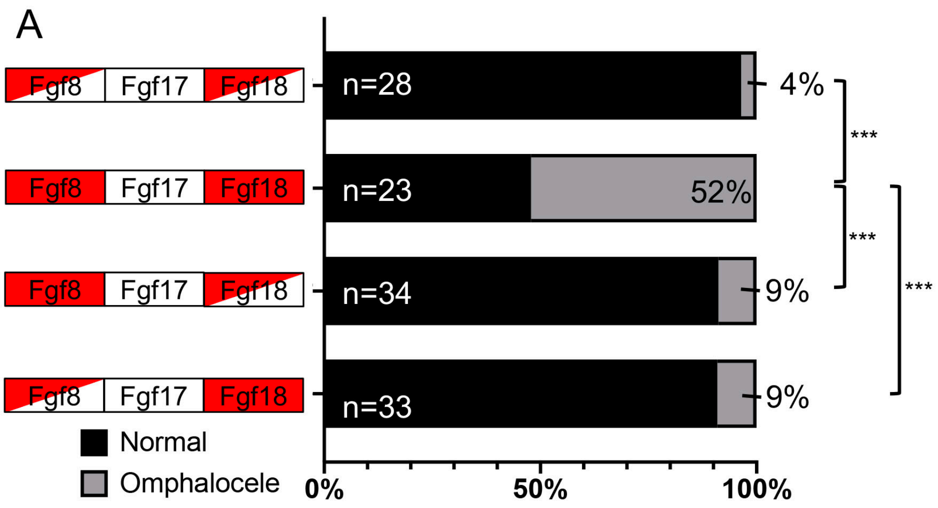

Figure S4. *Fgf17* does not rescue the omphalocele phenotype when copies of both *Fgf8* and *Fgf18* are inactivated. (A) Graphical representation of the incidence of omphalocele showing the percentage of embryos with and without omphalocele at E18.5. The total number of embryos is to the left of the bars and the percentage of embryos with omphalocele is on the right. Significance was found using a two-tailed Fisher's exact test. Boxes indicate genotype, see Fig. 2 for key.

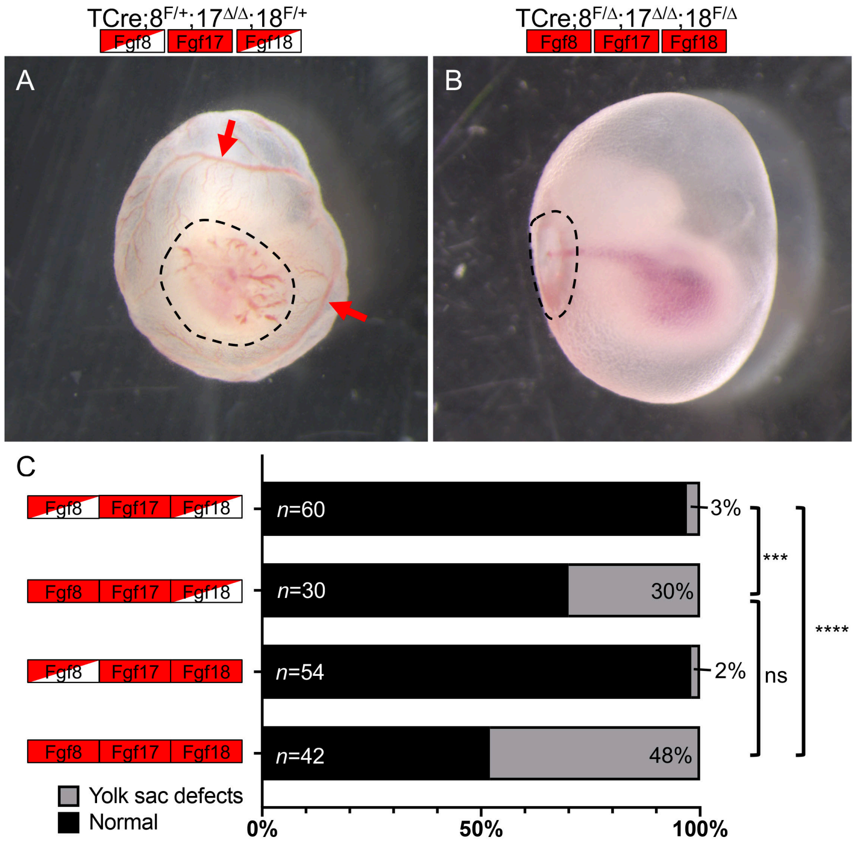

Figure S5. Loss of *Fgf8* results in an increased rate of yolk sac defects at E10.5. (A, B) E10.5 control (A) and triple mutant (B) embryo within the yolk sac. Mature yolk sac blood vessels indicated by arrows in A. Site of attachment to the chorion is indicated by a dashed circle. (C) Graphical representation of yolk sac defect incidence showing the percentage of embryos with and without yolk sac defects at E10.5, as determined by defective yolk sac remodeling and poor chorioallantoic fusion. The total number of embryos is to the left of the bars and the percentage of embryos with omphalocele is on the right. Significance was determined using a two-tailed Fisher's exact test. Boxes indicate genotype, see Fig. 2 for key.

Development • Supplementary information

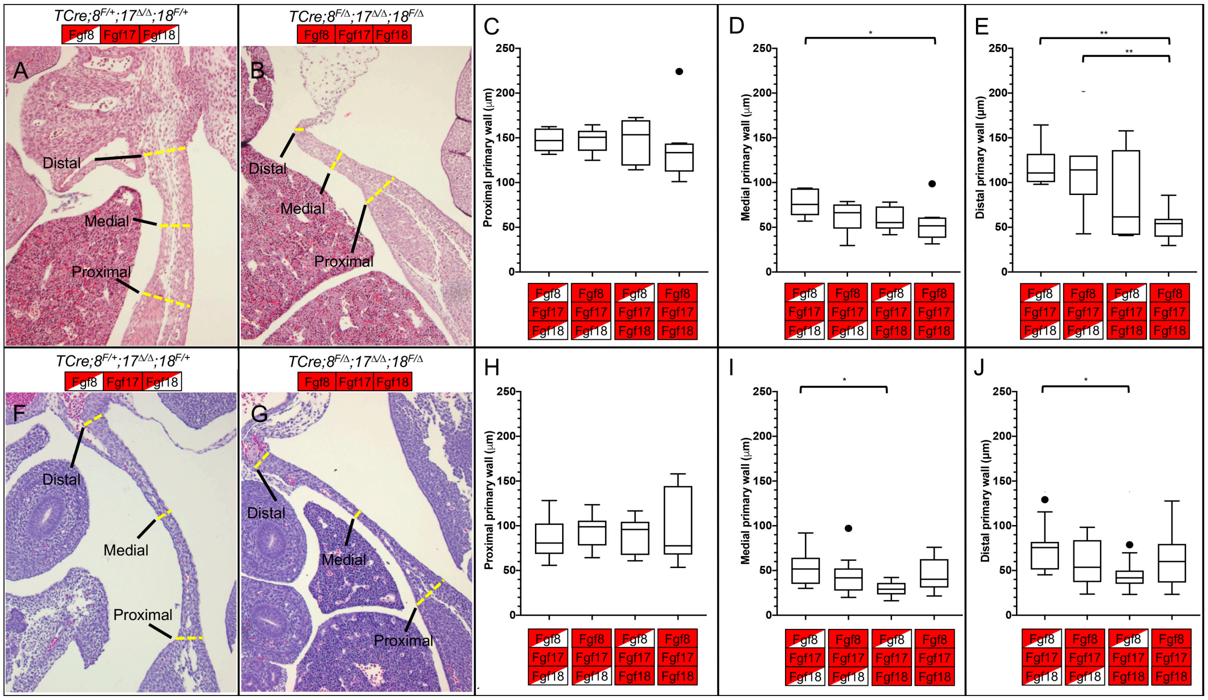

Figure S6. Morphometric analysis of sections at E13.5 and E12.5 reveals changes in the primary VW of *Fgf8* subfamily mutants. (A, B) Transverse section of E13.5 control (A) and triple mutant (B) at the interlimb region. (C-E) Tukey box-plots of the thickness of the E13.5 primary VW at the indicated positions (shown in A,B): proximal (C); medial (D); distal (E). Control *n*= 8; TCre;*Fgf8*$^{f/\Delta}$*,17*$^{\Delta/\Delta}$*,18*$^{f/+}$ *n*=7; TCre;*Fgf8*$^{f/+}$*,17*$^{\Delta/\Delta}$*,18*$^{f/\Delta}$ *n*=8; triple mutant *n*=9 (F,G) Transverse section of E12.5 control (F) and triple mutant (G) at the interlimb region. (H-I) Tukey box plots of the thickness of the E12.5 primary VW at the at the indicated positions (shown in F ,G): proximal proximal (H), medial (I) and distal (J) positions. Control *n*= 12; TCre;*Fgf8*$^{f/\Delta}$*,17*$^{\Delta/\Delta}$*,18*$^{f/+}$ *n*=10; TCre;*Fgf8*$^{f/+}$*,17*$^{\Delta/\Delta}$*,18*$^{f/\Delta}$ *n*=11; triple mutant *n*=11. Outliers in box-plots are plotted individually. Significance was found using a post-hoc Tukey-Kramer test. Boxes indicate genotype, see Fig. 2 for key.

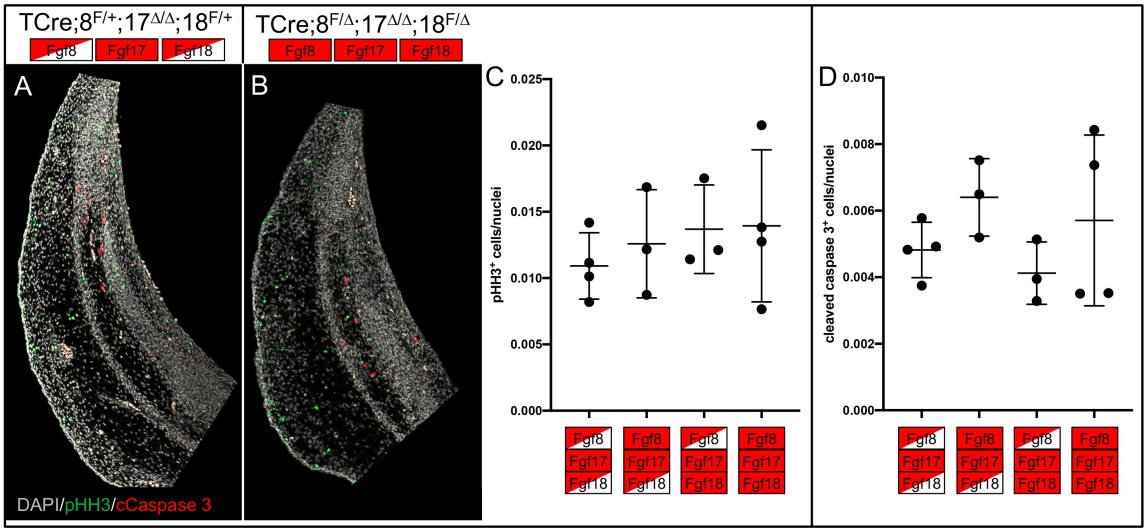

Figure S7. Cell proliferation and cell death in the secondary VW are unchanged in *Fgf8* subfamily mutants. (A,B) Max projection of 5 z-stacks from control (A) and triple mutant (B) transverse sections of the Secondary VW labelled with anti-pHH3 (green), anti-cleaved caspase 3 (red) and DAPI (white). (C) Ratio of pHH3 labelled cells to total nuclei. (D) Ratio of cleaved-caspase 3 to total nuclei. In C and D, data are plotted individually, mean±s.d. shown, a post-hoc Tukey-Kramer test applied. Control *n*=4; TCre;*Fgf8*$^{f/\Delta}$*,17*$^{\Delta/\Delta}$*,18*$^{f/+}$ *n*=3; TCre;*Fgf8*$^{f/+}$*,17*$^{\Delta/\Delta}$*,18*$^{f/\Delta}$ *n*=3; triple mutant *n*=4. Boxes indicate genotype, see Fig. 2 for key.

Development • Supplementary information

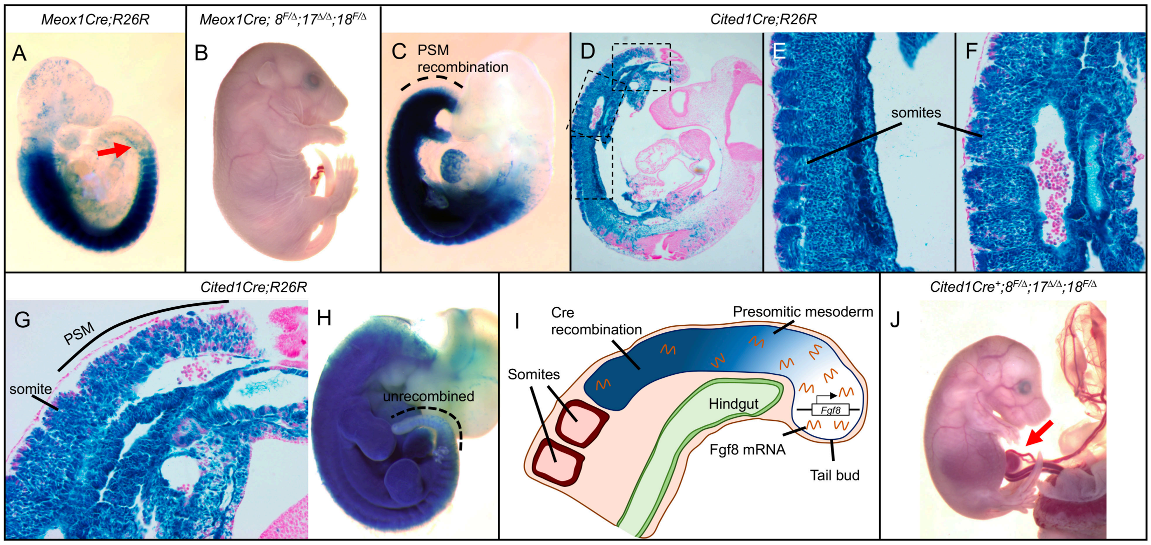

Figure S8. Meox1Cre recombines in the somites and Cited1Cre recombines in the PSM and the somites. (A) Recombination pattern of *Meox1Cre;R26R* E9.5 embryo, showing progressive somitic recombination (caudal-most somite indicated with arrow). (B) E18.5 *Meox1Cre;Fgf8$^{f/\Delta}$;Fgf17$^{\Delta/\Delta}$;Fgf18$^{f/\Delta}$* embryo. (C-H) Recombination pattern of Cited1Cre*;R26R*, tamoxifen injected at E7.5 and E8.5. (see Materials and Methods). (C) Recombination pattern of E9.5 Cited1Cre*;R26R*, embryo. D) Sagittal section of *Cited1Cre;R26R*, with higher magnifications of boxed regions shown in E-G. (H) Recombination pattern of E11.5 Cited1Cre*;R26R*, embryo. (I) Diagram showing the mechanism of perdurance of *Fgf8* mRNA in the PSM in Cited1Cre*;Fgf8$^{f/\Delta}$;Fgf17$^{\Delta/\Delta}$;Fgf18$^{f/+}$* and Cited1Cre;*Fgf8$^{f/\Delta}$;Fgf17$^{\Delta/\Delta}$;Fgf18$^{f/\Delta}$* embryos. (J) E15.5 Cited1Cre triple mutant embryo, showing omphalocele (arrow).

Development • Supplementary information

Table S1. Genotypes of experimental crosses used in this study.

Experimental cross			Experimental genotype*	Frequency	Control genotypes*	Frequency
Fgf8$^{F/F}$;17$^{\Delta/\Delta}$;18$^{F/F}$	X	TCre$^{Tg/Tg}$;Fgf8$^{\Delta/+}$;17$^{\Delta/\Delta}$;18$^{\Delta/+}$	TCre;Fgf8$^{F/\Delta}$;17$^{\Delta/\Delta}$;18$^{F/\Delta}$	1:4	**TCre;Fgf8$^{F/+}$;17$^{\Delta/\Delta}$;18$^{F/+}$**<br>TCre;Fgf8$^{F/\Delta}$;17$^{\Delta/\Delta}$;18$^{F/+}$<br>TCre;Fgf8$^{F/+}$;17$^{\Delta/\Delta}$;18$^{F/\Delta}$	1:4
Fgf8$^{F/F}$;17$^{+/+}$;18$^{F/F}$	X	TCre$^{Tg/Tg}$;Fgf8$^{\Delta/+}$;17$^{+/+}$;18$^{\Delta/+}$	TCre;Fgf8$^{F/\Delta}$;17$^{+/+}$;18$^{F/\Delta}$	1:4	**TCre;Fgf8$^{F/+}$;17$^{+/+}$;18$^{F/+}$**<br>TCre;Fgf8$^{F/\Delta}$;17$^{+/+}$;18$^{F/+}$<br>TCre;Fgf8$^{F/+}$;17$^{+/+}$;18$^{F/\Delta}$	1:4
Fgf8$^{F/F}$;17$^{\Delta/\Delta}$;18$^{+/+}$	X	TCre$^{Tg/Tg}$;Fgf8$^{\Delta/+}$;17$^{\Delta/\Delta}$;18$^{+/+}$	TCre;Fgf8$^{F/\Delta}$;17$^{\Delta/\Delta}$;18$^{+/+}$	1:2	**TCre;Fgf8$^{F/+}$;17$^{\Delta/\Delta}$;18$^{+/+}$**	1:2
Fgf8$^{+/+}$;17$^{\Delta/\Delta}$;18$^{F/F}$	X	TCre$^{Tg/Tg}$;Fgf8$^{+/+}$;17$^{\Delta/\Delta}$;18$^{\Delta/+}$	TCre;Fgf8$^{+/+}$;17$^{\Delta/\Delta}$;18$^{F/\Delta}$	1:2	**TCre;Fgf8$^{+/+}$;17$^{\Delta/\Delta}$;18$^{F/+}$**	1:2
Fgf8$^{F/F}$;17$^{+/+}$;18$^{+/+}$	X	TCre$^{Tg/Tg}$;Fgf8$^{\Delta/+}$; 17$^{+/+}$;18$^{+/+}$	TCre;Fgf8$^{F/\Delta}$; 17$^{+/+}$;18$^{+/+}$	1:2	**TCre;Fgf8$^{F/+}$; 17$^{+/+}$;18$^{+/+}$**	1:2
Fgf8$^{+/+}$;17$^{\Delta/\Delta}$;18$^{+/+}$	X	TCre$^{Tg/Tg}$;Fgf8$^{+/+}$; 17$^{\Delta/\Delta}$;18$^{+/+}$	TCre;Fgf8$^{+/+}$; 17$^{\Delta/\Delta}$;18$^{+/+}$	1	**N/A**	N/A
Fgf8$^{+/+}$;17$^{+/+}$;18$^{F/F}$	X	TCre$^{Tg/Tg}$;Fgf8$^{+/+}$;17$^{+/+}$;18$^{\Delta/+}$	TCre;Fgf8$^{+/+}$;17$^{+/+}$;18$^{F/\Delta}$	1:2	**TCre;Fgf8$^{+/+}$;17$^{+/+}$;18$^{F/+}$**	1:2
Fgf8$^{F/F}$;17$^{\Delta/\Delta}$;18$^{F/F}$	X	Cited1Cre^{Tg};Fgf8$^{\Delta/+}$;17$^{\Delta/\Delta}$;18$^{\Delta/+}$	Cited1Cre;Fgf8$^{F/\Delta}$;17$^{\Delta/\Delta}$;18$^{F/\Delta}$	1:8	**Cited1Cre;Fgf8$^{F/+}$;17$^{\Delta/\Delta}$;18$^{F/+}$**<br>Cited1Cre;Fgf8$^{F/\Delta}$;17$^{\Delta/\Delta}$;18$^{F/+}$<br>Cited1Cre;Fgf8$^{F/+}$;17$^{\Delta/\Delta}$;18$^{F/\Delta}$	1:8
Fgf8$^{F/F}$;17$^{\Delta/\Delta}$;18$^{F/F}$	X	Meox1$^{Cre/+}$;Fgf8$^{\Delta/+}$;17$^{\Delta/\Delta}$;18$^{\Delta/+}$	Meox1$^{Cre/+}$;Fgf8$^{F/\Delta}$;17$^{\Delta/\Delta}$;18$^{F/\Delta}$	1:8	**Meox1$^{Cre/+}$;Fgf8$^{F/+}$;17$^{\Delta/\Delta}$;18$^{F/+}$**<br>Meox1$^{Cre/+}$;Fgf8$^{F/\Delta}$;17$^{\Delta/\Delta}$;18$^{F/+}$<br>Meox1$^{Cre/+}$;Fgf8$^{F/+}$;17$^{\Delta/\Delta}$;18$^{F/\Delta}$	1:8

The experimental cross is always written 'female x male'.
Δ, 'deletion' or null; +, wild-type.
The most wild-type control from a genetic cross is written in bold.
The genotypes of Cre-negative offspring from crosses using Meox1Cre and Cited1Cre are not shown here.

Development • Supplementary information
