# Supplementary material for: The Fgf8 subfamily (Fgf8, Fgf17 and Fgf18) is required for closure of the embryonic ventral body wall
Source: Development. 2020 Oct 19;147(21):dev189506. doi: 10.1242/dev.189506 (PMC7595690; doi:10.1242/dev.189506)
Supplement: Reviewer comments [file develop-147-189506_review_history.pdf]

The Fgf8 subfamily (Fgf8, Fgf17 and Fgf18) is required for closure of the embryonic ventral body wall

Michael Boylan, Matthew J. Anderson, David M. Orntiz and Mark Lewandoski
DOI: 10.1242/dev.189506

Editor: Sally Dunwoodie

Review timeline

Original submission:	17 February 2020
Editorial decision:	11 March 2020
First revision received:	20 August 2020
Accepted:	28 August 2020

Original submission

First decision letter

MS ID#: DEVELOP/2020/189506

MS TITLE: The Fgf8 subfamily (Fgf8, Fgf17 and Fgf18) is required for closure of the embryonic ventral body wall

AUTHORS: Mark Lewandoski, Michael Boylan, Matthew Anderson, and David Orntiz

I have now received all the referees reports on the above manuscript, and have reached a decision. The referees' comments are appended below, or you can access them online: please go to BenchPress and click on the 'Manuscripts with Decisions' queue in the Author Area.

The overall evaluation is positive and we would like to publish a revised manuscript in Development, provided that the referees' comments can be satisfactorily addressed. Please attend to all of the reviewers' comments (except for comment 3 of Reviewer 2) in your revised manuscript and detail them in your point-by-point response. If you do not agree with any of their criticisms or suggestions explain clearly why this is so.

Reviewer 1

Advance summary and potential significance to field

The mechanisms regulating ventral body closure during development are complex and still largely unknown.
This work adds the novel and important information that a family of FGF (8, 17 and 18) is required for this process to occur. This may have also important implications in human pathology.

Comments for the author

The work by Boylan and colleagues report the novel and interesting observation that three FGF family mambers are required for body wall closure in the mouse. Because of the partial redundancy of the three FGFs a number of compound mutant embryos are generated and the resulting phenotype analysed in detail.

© 2020. Published by The Company of Biologists under the terms of the Creative Commons Attribution License (https://creativecommons.org/licenses/by/4.0/).

Overall this is an excellent paper, well written and easy to read despite the complexity of the various crosses.
The experiments are well planned and the results are of high quality. The discussion points to future directions in the field. Overall I have no major criticisms and recommend publication.

Minor comment: On page 12, line 3 the authors state :"Although spontaneous abortions due to tamoxifen administration prevented recovery of E18.5 embryos, we reliably recovered E15.5 embryos at Mendelian ratios." I am surprised that despite co-administration of progesterone abortion seems the unavoidable outcome. Although abortion may occur after tamoxifen administration, I am surprised that no animals were born or reached E18.5. Authors may want to discuss this issue.

<u>Reviewer 2</u>

Advance summary and potential significance to field

The manuscript by Boyle and colleagues examines the role of FGF8 family members 8,17, and 18 in the closure of the ventral body wall and its relevance to omphalocele. The research revealed a novel role for the Fgf8 ligands in promoting an adequate number of progenitor cells in the somites and influencing the migration of cells of the secondary body wall towards the midline. Fgf8, 17, and 18 play unique and overlapping roles contributing to the formation of the ventral body. wall. Thus, the findings emphasize the multifactorial etiology of birth defects of the ventral body wall.

Comments for the author

The manuscript by Boyle and colleagues examines the role of FGF ligands 8, 17, and 18 in ventral body wall formation. Using different CKO mouse and Cre-recombinase lines, the authors interrogated the spatial and time requirements of the different ligands for the ventral body wall. The authors conclude that an additive genetic effect of FGF8,17 and 18 is required for the closure of the ventral body wall. The subject of study is relevant to understanding the etiology of congenital malformations affecting the body wall and thus of interest to the journal's readership. While the research was carefully performed, authors should address the following issues before the manuscript can be recommended for publication.

1. What is the expression of FGF receptors concerning the FGF ligands analyzed in the manuscript? Are FGF 8 and 17 present in the primary body wall at E.11? In situ hybridization on sections of the later stages presented in figure 1 will help to discern the expression pattern of FGFs and FGF receptors in the primary body wall and developing secondary body wall.
2. Is the histological appearance of the primary body wall affected in triple mutants or any of the mutants analyzed?
3. Are muscle cells isolated from the triple mutants secondary body wall capable of migrating in vitro? Could treatment with FGF ligands affect the migration of the cells in vitro?
4. Fgf18 provides directional cues for cartilage formation in developing respiratory tract airways. Are FGF8,17 and 18 required for the directional movement of the secondary body wall cells to the midline? Is the primary body wall a source of FGF during the migration of secondary body wall into the midline?
5. Based on the skeletal defects observed in the triple mutants, is the thoracic body wall affected? Is the Sternum appropriately formed?
6. In Figure 7, higher magnification images will facilitate to distinguish expression pattern of Msgn1 and Unx4.1.
7. Is there evidence that variants for FGF8, FGF17, or FGF18 are associated with body wall defects in humans?

<u>Reviewer 3</u>

Advance summary and potential significance to field

DEV189506 describes about differential FGF functions regulating the closure of ventral body wall.

© 2020. Published by The Company of Biologists under the terms of the Creative Commons Attribution License (https://creativecommons.org/licenses/by/4.0/).

The authors group long investigated on FGF functions using unique conditional mutant mouse series. They conducted conditional KO analysis on different FGF genes for body wall closure regulation. They found FGF18 is required for somites in contrast to FGF8/17 for pre somatic mesoderm. Regulation of body wall closure is an important topic not only for organogenesis field but also necessary to understand the corresponding birth defects.

Comments for the author

Comments
The work is offers essential info to birth defect researches. It describes different members of FGF genes contributing to the onset of complex birth detect; body wall of abnormality.
It can be also noted that the work is rather descriptive.

This study is the first showing a role for FGF ligands in VW closure, and they demonstrated a gene dosage effect on the incidence of omphalocele.

They present evidences for genetic redundancy between members of the Fgf subfamily. By using several Cre lines, they determined the spatial and temporal requirements for such genes.
The discussion about FGF redundancy would be one of the highlight-paper topics. However, the current finding does not offer nor indicate enough future directions to study such FGF redundancy. They need to describe THE FUTURE RESARCH DIRECTION BASED ON THE NOVELITY OF CURRENT FINDINGS. Such emphasized description should guide Development readers for future direction and perspectives.

Kyphosis caused by a loss of Fgf18 increases intra-abdominal pressure, contributing to the phenotype.
Kyphosis has been proposed as a contributory factor in several other mouse models of omphalocele
Discussion about the possible correlation of kyphosis and intra-abdominal pressure will be useful for pathogenesis discussion.

The regulation of Msgn1 expression and its domain would be potentially intriguing but not enough to offer concrete set of future research directions.

First revision

<u>Author response to reviewers' comments</u>

We are grateful to you and the reviewers for the time and attention spent on this manuscript. All the reviewer's criticisms and advice have improved the manuscript. Below, we attend to each of the reviewer's comments, addressing each one (with the exception of comment 3 of reviewer 2, per your instructions). In total, we have added two new figures (Figure 1S and Figure S6 and replaced panels A, B, F and G in Figure 7 per Reviewer's 2 instructions. In our responses below, we explicitly describe textual changes to the draft. Also, we made many small textual changes throughout the paper in order to meet the word-limit requirements of the journal. We do not list these small changes here.

Reviewer 1. We thank the reviewer for her/his opinion: "Overall this is an excellent paper, well written and easy to read despite the complexity of the various crosses. The experiments are well planned and the results are of high quality."

1) Regarding spontaneous abortions due to tamoxifen administration (despite progesterone administration), the reviewer wrote : "I am surprised that no animals were born or reached E18.5…. Authors may want to discuss this issue."

We apologize for the confusion from our inaccurate description in our initial draft. We initially attempted to recover embryos at E18.5, and while some pregnancies were maintained until E18.5,

© 2020. Published by The Company of Biologists under the terms of the Creative Commons Attribution License (https://creativecommons.org/licenses/by/4.0/).

we also had a lot of aborted pregnancies (regardless of embryonic genotype). Due to the relatively low frequency of recovering each genotype (1/8) and the relatively high numbers required needed for statistical analyses, we needed to recover embryos more efficiently. Thus we modified our protocol to recover embryos at E15.5, and avoided the late stage loss of pregnancy. It is unclear as to why progesterone does not have as much of a protective effect as we had hoped, but we speculate that due to the early timing of induction (E7.5 and E8.5), any protection might have worn off by the end of gestation. Although we note that there are not any good, controlled published data showing that progesterone protects litters from Tam- induced toxicity. Papers mentioning the application of progesterone do so anecdotally (Dev Dyn 235:2376-85, Methods Enzymol. 477: 153–181, Dev Dyn 235:2603-12); this paucity of quantitative data includes accounts that describe progesterone *not* protecting mouse litters from Tam-toxicity (Transgenic Res 24:1065–1077).

We changed our text (page 12, lines 10-13) to “Spontaneous abortions due to tamoxifen administration prevented recovery of E18.5 embryos often enough to impede our efforts. However, we reliably recovered E15.5 embryos at Mendelian ratios, which allowed analysis because the phenotype was evident by E13.5 (Fig. 3).”

Reviewer 2. We thank the reviewer for her/his opinion: “The subject of study is relevant to understanding the etiology of congenital malformations affecting the body wall and thus of interest to the journal's readership. While the research was carefully performed, authors should address the following issues…”

1) What is the expression of FGF receptors concerning the FGF ligands analyzed in the manuscript? Are FGF 8 and 17 present in the primary body wall at E.11?

To answer where and when the relative FGF receptor genes are expressed, we have added the following text to our previous Discussion paragraph (now page 16, lines 4-12), where we had previously discussed the *Fgfr* mutant omphalocele publication:

“*Fgfr1* transcripts are detected in the PSM and both *Fgfr1* and *Fgfr2* genes are expressed in the somites and LPM at E8.5 and E9. (Orr-Urtreger et al., 1991; Wahl et al., 2007). Thus, the expression domains of these receptor genes are consistent with our model (Fig. 8). At E11.5 - E13.5, these receptor genes are also expressed in the VW itself; *Fgfr1* in a broad domain and *Fgfr2* transcripts are detected in VW subsets (Nichol et al., 2011; Orr-Urtreger et al., 1991; Peters et al., 1992). These later expression domains of the *Fgfr* genes suggest that FGF signaling may act at multiple locations to close the VW, a complexity similar to the role of FGF signaling in limb development (Benazet and Zeller, 2009; Jin et al., 2018).”

Regarding expression of the FGF ligand genes in the primary and secondary walls at E11.5, we think there is negligible expression (although, of course, it is impossible to prove a negative). As mentioned in the first paragraph of the Results (in both the original and current drafts), the colorimetric WISH analysis in Figure 1, was overstained to “reveal domains of low gene expression.” (line 17, page 5). To emphasize this, we have added, at the end of page 5- beginning of page 6, “The data in Figure 1 indicates that there is either no or very low expression of these ligand genes in the primary VW or secondary VW layers themselves.”

Furthermore, we have also generated new hybridization chain reaction (HCR) analysis for each of the these three Fgf ligand genes and have added this as Figure S1. On page 6, lines 1-4, we added the text . “To address this directly, we generated E11.5 in situ hybridization chain reaction (HCR) data for each of the three FGF ligand genes (Fig. S1). These analyses confirmed our observation that there is little to no expression within the VW itself.”

2) Is the histological appearance of the primary body wall affected in triple mutants or any of the mutants analyzed?

We have included a new supplementary figure (Figure S6) and additional text discussing how the histology of the primary VW was affected in the different genotypes. In sum, the primary VW is thinner in triple mutants than in controls, at E13.5, but not but at E12.5 . On page 9, lines 9-10,

© 2020. Published by The Company of Biologists under the terms of the Creative Commons Attribution License (https://creativecommons.org/licenses/by/4.0/).

discussing E13.5 stages, we added We found that while the length of the primary VW was unchanged (Fig. 4G), the primary VW was thinner in triple mutants (Fig. S6A-E). At the bottom of page 9, discussing E12.5 stages, we added: “The thickness of the primary VW was also unaffected at this stage in triple mutants (Fig. S6F-J), though the primary VW is thinner in TCre;*Fgf8*$^{f/+}$;*Fgf17*$^{\Delta/\Delta}$;*Fgf18*$^{f/\Delta}$ embryos (Fig. S6I,J).”

4) Fgf18 provides directional cues for cartilage formation in developing respiratory tract airways. Are FGF8,17 and 18 required for the directional movement of the secondary body wall cells to the midline? Is the primary body wall a source of FGF during the migration of secondary body wall into the midline?

Given our data in Figs. 1 and S1, we speculate this is not the case (i.e. the Fgf genes don’t seem to be expressed in the primary wall/midline. But we thank the reviewer for thinking of this (as well as asking for information regarding the Fgf receptor gene expression domains). This prompted us to add to the Discussion (page 16, lines 7-15: “At E11.5 - E13.5, these receptor genes are also expressed in the VW itself; *Fgfr1* in a broad domain and *Fgfr2* transcripts are detected in VW subsets (Nichol et al., 2011; Orr-Urtreger et al., 1991; Peters et al., 1992).
These later expression domains of the *Fgfr* genes suggest that FGF signaling may act at multiple locations to close the VW, a complexity similar to the role of FGF signaling in limb development (Benazet and Zeller, 2009; Jin et al., 2018). If FGFs provide directional cues for secondary VW migration at these later stages, as they do during migration of tracheal cartilage (Elluru et al., 2009), our data suggest they may be encoded by *Fgfs* outside the *Fgf8* subfamily.”

5) Based on the skeletal defects observed in the triple mutants, is the thoracic body wall affected? Is the Sternum appropriately formed?

On page 6, lines 28-29, we added “This failure to close the ventral body was always abdominal and never affected the thoracic body wall.“ On page 7, lines 16-17, discussing the skeletal defects, we now added the sentence “Although the sternum appeared kinked due to the rib abnormalities, it had fused correctly (data not shown).”

6) Figure 7, higher magnification images will facilitate to distinguish expression pattern of Msgn1 and Unx4.1.

We have replaced our original images in figure with higher magnification images.

7) Is there evidence that variants for FGF8, FGF17, or FGF18 are associated with body wall defects in humans?

On page pg 16, lines 20-24, we added the text “In humans, omphalocele is reported in two case studies of Aperts syndrome (Ercoli et al., 2014; Herman and Siegel, 2010), which is caused by Fgfr2 mutations, but is otherwise known as a craniosynostosis pathology (Armand et al., 2019). Otherwise, there are no reports of mutations in *Fgf* ligands or receptors causing VW defects in humans, possibly due to genetic redundancy or embryonic lethality.”

Reviewer 3 We thank the reviewer for her/his opinion:” The work is offers essential info to birth defect researches.”

1) The reviewer expressed the criticism: “…the current finding does not offer nor indicate enough future directions to study such FGF redundancy. They need to describe THE FUTURE RESARCH DIRECTION BASED ON THE NOVELITY OF CURRENT FINDINGS.

Regarding FGF redundancy we have added two ideas in the Discussion section in the following text (page 16, lines 12-18): “If FGFs provide directional cues for secondary VW migration at these later stages, as they do during migration of tracheal cartilage (Elluru et al., 2009), our data suggest they may be encoded by Fgfs outside the Fgf8 subfamily. This later FGF signaling step may be upstream of MEK1 and MEK2 function, which is required at these stages to close the VW (Boucherat et al., 2014). Future work will determine if MEK 1/2 kinases function downstream of the FGF8 subfamily in the PSM and somites.”

© 2020. Published by The Company of Biologists under the terms of the Creative Commons Attribution License (https://creativecommons.org/licenses/by/4.0/).

Otherwise, we added at the end of the paragraph discussing kyphosis (page 17, lines 6-7): "Therefore, the causality between kyphosis and omphalocele is unclear and warrants future study." Also, in the penultimate paragraph (straddling pages 17-18) "Additionally, we suggest that FGF signals do not provide directional cues *per se* for the secondary VW lineage, but are generally required for migration, as is the case for FGF signaling in limb and axis extension (Benazeraf et al., 2010; Gros et al., 2010; Lewandoski and Mackem, 2011). This idea is the focus of our future work."

2) The review suggested that "Discussion about the possible correlation of kyphosis and intra-abdominal pressure will be useful for pathogenesis discussion."

We have taken our previous paragraph on kyphosis in the Discussion and elaborated on our ideas concerning this (the paragraph straddles pages 16 and 17) "Kyphosis has been proposed as a causal factor in several mouse models of omphalocele (Boucherat et al., 2014; Kakizaki et al., 2015) with the rationale that a malpositioned spine could reduce the volume of the abdominal cavity and increase intraabdominal pressure, forcing the viscera out through the umbilicus. We observed that triple mutants, compared to $TCre^{+};Fgf8^{f/+};Fgf17^{\Delta/\Delta};Fgf18^{f/\Delta}$ embryos, have both a greater rate of omphalocele and more severe kyphosis, suggesting spinal defects may exacerbate VW defects. However, it is not the case that kyphosis is the primary cause, because omphalocele occurs in $TCre^{+};Fgf8^{f/+};Fgf17^{\Delta/\Delta};Fgf18^{f/+}$ mutants, which lack kyphosis, demonstrating that VW defects occur with a normal spine. Furthermore, in human patients, once the omphalocele is repaired, the volume of the abdominal cavity and the posture of the spine both recover (Nagaya et al., 2000), suggesting omphalocele may cause spine defects. Therefore, any causality between kyphosis and omphalocele is unclear and warrants future study."

<u>Second decision letter</u>

MS ID#: DEVELOP/2020/189506

MS TITLE: The Fgf8 subfamily (Fgf8, Fgf17 and Fgf18) is required for closure of the embryonic ventral body wall

AUTHORS: Mark Lewandoski, Michael Boylan, Matthew Anderson, and David Orntiz
ARTICLE TYPE: Research Article

I am happy to tell you that your manuscript has been accepted for publication in Development, pending our standard ethics checks.

© 2020. Published by The Company of Biologists under the terms of the Creative Commons Attribution License (https://creativecommons.org/licenses/by/4.0/).
